# Therapeutic targeting of RAGE/STAT3 signaling abrogates S100A7-driven breast tumorigenicity and immune suppression

**DOI:** 10.1186/s13058-026-02281-0

**Published:** 2026-04-29

**Authors:** Pratyusha Ghanta, Ajeet K. Verma, Cho-Hao Lin, Manish Charan, Ganesh R. Koshre, Shashwat Agarwal, Jonathan Adorno, Ayush Arpit Garg, Tanisha Mukherjee, Wayne O. Miles, Jonathan W. Song, Sanjay Mishra, Ramesh K. Ganju

**Affiliations:** 1 Department of Pathology, College of Medicine, The Ohio State University, Columbus, OH 43210 USA; 2 Comprehensive Cancer Center, The Ohio State University, Columbus, OH 43210 USA; 3 Department of Cancer Biology and Genetics, The Ohio State University, Columbus, OH 43210 USA; 4 Department of Mechanical and Aerospace Engineering, The Ohio State University, Columbus, OH 43210 USA

**Keywords:** S100A7, RAGE, STAT3, SERPIN-E1, And TNBC

## Abstract

Metastatic breast cancer, particularly triple-negative breast cancer (TNBC), remains a major clinical challenge due to its aggressive behavior and limited therapeutic options. The molecular pathways that drive tumor progression while simultaneously promoting immune evasion are not well defined. Here, we identify S100A7/RAGE signaling as a central oncogenic and immunomodulatory axis that drives TNBC tumorigenicity, metastasis, and immune suppression. In this study, we have shown that S100A7/RAGE signaling plays a vital role in driving invasive TNBC tumorigenicity by activating Stat3 and elevating Serpin-E1 expression. Furthermore, we revealed that combinatorial treatment of Stat3 and RAGE inhibitors synergistically inhibits the tumorigenicity of S100A7-expressing TNBC cells in-vitro by suppressing AKT/MAPK signaling and mitigates the tumor burden, especially distant metastasis in-vivo using TNBC cell lines and a conditional mammary gland-specific S100A7 overexpression bitransgenic mouse model. Moreover, our study also shed light on the dynamic interplay between the S100A7/RAGE/Stat3 signaling pathway and immune cell infiltration within the tumor microenvironment. Remarkably, our findings underscored the potential of combined inhibition to enhance anti-tumor immune responses, fostering the infiltration of anti-tumor MHCII^high^ and iNOS^+^ macrophages and activated effector CD8^+^ tumor-infiltrating T cells. Moreover, CD8^+^ T cells depletion abrogates the therapeutic benefits of RAGE/Stat3 inhibition by restoring the tumor growth in S100A7 overexpression mice. Importantly, we discovered that S100A7/RAGE signaling modulates anti-tumor macrophage phenotypes by regulating Serpin-E1 expression on TNBC cells. S100A7 regulates Serpin-E1 gene expression by enhancing the direct binding of pStat3 (Ser727) to its promoter. Notably, neutralization of Serpin-E1 in combination with RAGE and Stat3 inhibition increased iNOS expression in macrophages in-vitro and significantly enhanced overall anti-metastatic efficacy in-vivo. Importantly, we found that increased co-expression of S100A7 and Serpin-E1 correlated with worse prognoses in TNBC patients, whereas the presence of iNOS or MHCII genes improved the overall prognosis, especially basal and immunomodulatory subtypes, which also underscores the clinical relevance of targeting these signaling molecules as a promising translational therapeutic strategy for TNBC. Overall, these findings provide new avenues for therapeutic interventions in metastatic TNBC.

**Graphical abstract:**

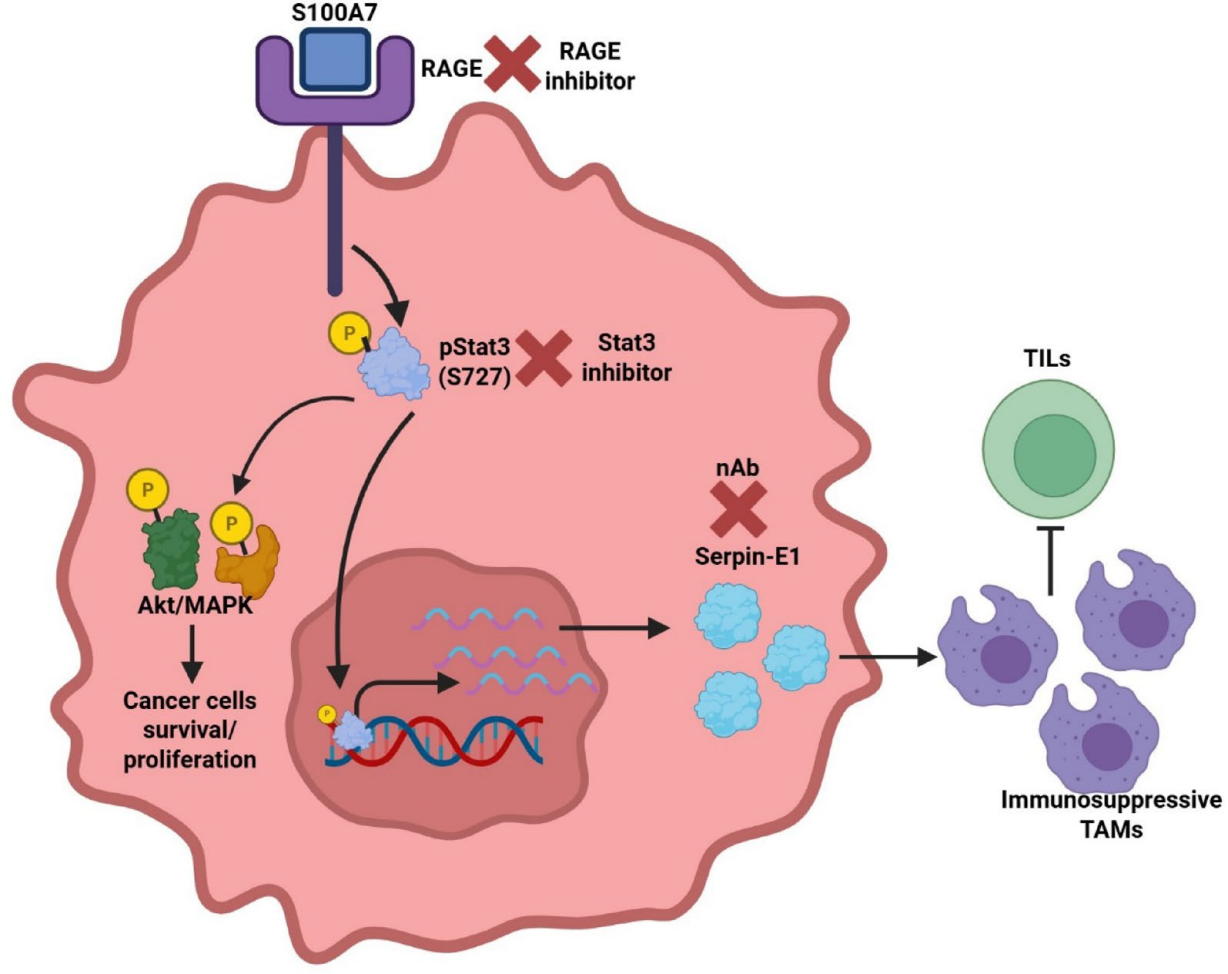

**Supplementary Information:**

The online version contains supplementary material available at 10.1186/s13058-026-02281-0.

## Introduction

Metastatic breast cancer, particularly Triple-Negative Breast Cancer (TNBC), presents significant treatment challenges due to its aggressive nature and limited targeted therapeutic options [1]. The lack of specific molecular targets renders traditional therapeutic options, such as chemo or endocrine therapy and anti-HER2 therapy, ineffective for TNBC patients. Despite standard non-surgical treatments, including combination therapies and radiotherapy, exhibiting limited efficacy, TNBC remains associated with the highest mortality rates among breast cancer subtypes [1]. Thus, there is a critical need for the development of more effective and personalized treatments tailored to individual patients. Research focusing on understanding the mechanisms of metastasis and identifying novel therapeutic targets is essential to improve outcomes and quality of life for those with advanced breast cancer. In response to this clinical challenge, there has been a paradigm shift towards targeted therapies and immunotherapies, necessitating the identification of novel molecular targets to disrupt TNBC growth and metastasis while concurrently boosting the innate immune system [2, 3].

In our previous studies, we revealed that S100A7, a pro-inflammatory protein, binds to the receptor for advanced glycation endproducts (RAGE), thereby fostering breast tumor growth and metastasis [4]. This occurs by instigating an inflammatory tumor microenvironment (TME) by recruiting immunosuppressive myeloid cells [4–6]. Additionally, there have been reports, including our findings, indicating the overexpression of both S100A7 and RAGE in TNBC and metastatic breast cancers [4–7]. Our studies demonstrated that administering either a RAGE-neutralizing antibody or soluble RAGE effectively suppressed both breast tumor progression and metastasis using a S100A7 overexpressing (mS100a7a15) breast cancer mouse model [4]. More recently, the signal transducer and activator of transcription 3 (Stat3) has also gained attention as a crucial molecular target within the complex landscape of TNBC. Stat3 activation in tumor cells functions as a critical oncogenic mediator and transcriptional factor (TF) [8]. Elevated Stat3 activation has been associated with the aggressive nature of TNBC, promoting uncontrolled cell growth and dissemination to distant sites. Additionally, during the initial phases of breast carcinogenesis, Stat3 plays a key role in creating an immunosuppressive TME, facilitating tumor growth and metastasis [8]. Notably, the binding of the Stat3 TF to the S100A7 gene promoter drives S100A7 expression in breast cancer cells [9]. Furthermore, RAGE signaling has also been shown to activate Stat3 in cancer cells [10, 11]. However, the precise direct regulation of Stat3-mediated downstream signaling by the S100A7/RAGE axis in the context of metastatic breast cancer growth and metastasis through modulating the immune TME remains poorly understood. Recently, the aberrant high expression of Serpin-E1, also known as plasminogen activator inhibitor-1 (PAI-1), in TNBC has also emerged as a significant factor influencing breast TME [12, 13]. Serpin-E1, traditionally recognized for its role in fibrinolysis regulation, assumes a dual role by impacting the immune landscape in tumor tissue [14]. High expression levels of Serpin-E1 have been associated with the inhibition of M1-like anti-tumor macrophages, which are the critical components of the immune response against cancer [14]. Therefore, understanding the molecular mechanism that regulate Serpin-E1 expression and its effect on macrophage polarization may shed light on potential therapeutic strategies to disrupt this interaction and enhance anti-tumor immune responses in metastatic TNBC.

Here, our study established the potential crosstalk between S100A7/RAGE and Stat3/Serpin-E1 signaling in TNBC, especially through regulating immune cells function in TME of metastatic breast cancer. Moreover, our current study also determined the translational impact of combinatorial inhibition of Stat3 and RAGE to suppress the S100A7-mediated metastatic breast tumor growth and metastasis. Our study delineates the S100A7/RAGE-mediated regulation of Stat3/Serpin-E1 signaling in TNBC cells and how the combined pharmacological inhibition of this oncogenic signaling attenuates the tumorigenic effect of S100A7 on metastatic breast cancer. Ultimately, our research aims to provide a comprehensive framework for developing targeted interventions that inhibit cancer cell growth or metastasis and enhance the innate immune response in the challenging context of aggressive TNBC.

## Materials and methods

### Chemicals and reagents

#### Cell culture

Human TNBC cell lines (MDA-MB-231, and MDA-MB-468) and macrophage/monocytic cell line (CRL-9855) were obtained from ATCC. These cell lines were cultured and grown in DMEM medium supplemented with 10% FBS and 1% Penicillin/streptomycin solution. The CRL-9855 was cultured and grown in an IMDM medium supplemented with 10% FBS and 1% Penicillin/Streptomycin solution. S100A7-overexpressing MDA-MB-231 (231-S7OE) cells were generated in our lab by transfecting the MDA-MB-231 cell line with pIRES2-EGFP-hS100A7 using Lipofectamine-2000 reagent as per the manufacturer’s instructions, and stable S100A7 overexpression cells were screened using G418 selection (500 mg/mL). S100A7 knockdown cells were generated and maintained as described in our earlier study [5].

#### Cell viability assay

All TNBC cells were seeded in 96-well plates and grown for 24 h. At 70–80% confluency, these cells were serum starved for 4 h and then treated with different concentrations of Stat3 inhibitor (S3I-201; Selleckchem) and RAGE inhibitor (FPS-ZM1; Selleckchem) alone or in combination with their respective vehicle controls (DMSO) for 24 h, and cell viability was measured using the PrestoBlue Cell Viability Reagent (Thermo Fisher Scientific).

#### Cell migration and wound healing assays

TNBC cells (5X10^3^) in serum-free media were plated in an 8.0-µm pore size trans well insert (Costar) seeded in a 24-well plate. DMEM containing 5% FBS was added in the bottom chamber, whereas vehicle control, Stat3 inhibitor (IC_50_ values; 100 µM for 231-S7OE and MDA-MB-468 cells), RAGE inhibitor (IC_50_ values; FPS-ZM1, 25 µM for 231-S7OE cells, and 20 µM for MDA-MB-468 cells), and a combination of both inhibitors (CI < 1.0; showing maximum synergism) were added in serum-free media in the upper chamber (transwell insert). Cells were allowed to migrate overnight (~ 12 h), and cells on the bottom of the membrane were fixed and stained as described earlier [15]. The trans well inserts were washed 3 times with 1X PBS at both sides of the membrane. Several images have been captured to cover the migrated cells present on the surface of the trans-well membrane. Cell migration assays were also performed using CRL9855 cells (human macrophage) incubated in a control medium (IMDM) or 231-S7OE conditioned medium (CM), either supplemented with control IgG or Serpin-E1 nAb (300 ng/mL). CM were collected from S100A7-expressing breast cancer cells 24 h after completion of RAGE inhibitor, STAT3 inhibitor, or combination treatments to ensure that no residual inhibitors were present. These CM were then used for migration assays with CRL-9855 cells. For the wound healing assay, 231-S7OE and MDA-MB-468 cells were seeded in a 6-well plate and allowed to reach ~ 80% confluency, and then scratches were generated manually by the 200μL tips and bright field images were captured at initial time points as described earlier [15]. Next, these cells were serum starved and treated with VC, Stat3 inhibitor, RAGE inhibitor, and their combinations as described above for ~ 12 h. At the end of each treatment, images were captured again, and % would healing was measured as described previously [15].

#### Single cell migration experiment

24 h prior to seeding, 231-S100A7-OE cells grown in T-75 flasks were treated with growth media (DMEM + 10% FBS + 1% Pen-Strep) alone or with growth media supplemented with RAGE and Stat3 inhibitors, or thier combination as described above in migration assay. Subsequently, cells were seeded in the central chamber of a PDMS microdevice as described previously at 1X10^4^ cells/μL, and the media in all ports was replaced with serum-free growth media. Post 12 h of incubation (37 °C and 5.0% CO_2_), serum-free growth media supplemented with 100 ng/mL epidermal growth factor (EGF) replaced the old media in the top and bottom ports to promote cell migration from central to outer ports. Following this, time-lapse movies of the entire tracks within devices were acquired for 12 h using the Lionheart live imaging microscope at 4X magnification. During imaging, cells within devices were cultured at 37 °C and 5.0% CO_2_. These movies were analyzed using the MtrackJ plugin in Fiji to determine average cell speed, distance traveled, and displacement data.

#### Colony formation assay

1X10^3^ TNBC cells were seeded in a 6-well plate and grown for 24 h. After 24 h, cells were serum starved and treated with VC, Stat3 inhibitor, RAGE inhibitor, and their combination as described above. Next, the media containing these inhibitors was replaced with fresh complete DMEM media and grown for the next 5 days. At the end of the experiment, cells were stained with Giemsa stain, and colony-forming units were counted in each treatment group.

#### ChIP-qRT-PCR

MDA-MB-231 vector control (vector control) and S100A7-overexpressing (S100A7-OE) cells were cultured in 10% FBS, 1% penicillin–streptomycin, and 1% L-glutamine following standard ATCC protocols. Cells were fixed with 1% formaldehyde for 10 min, and crosslinking was quenched with glycine for 5 min. After washing with cold PBS, the cells were resuspended in a lysis buffer. Chromatin was sheared to 100–500 bp using a sonicator at 38% amplitude with 20-s “ON” and 10-s “OFF” cycles for 5 min. pStat3 (Ser727; CST #9134) antibody was coupled to magnetic beads (#9006S), washed trice, and incubated with solubilized chromatin. Chromatin complexes were pulled down with a magnet, washed, and eluted. Crosslinking was reversed, followed by RNase I and proteinase K treatment. Immunoprecipitated DNA was extracted using phenol–chloroform-isoamyl alcohol precipitation. The eluted DNA concentration was measured and subjected to qRT-PCR analysis for SERPINE1 and respective controls. The ChIP qRT-PCR data were analyzed as described previously [16, 17].

### Mouse models and tumor cells injection

All mice were kept in The Ohio State University’s animal facility in compliance with the guidelines and protocols approved by the OSU-IACUC. All animal procedures were performed with approval from the University Laboratory Animal Resources (ULAR) at OSU. We performed our in-vivo studies using 6-week-old mammary gland-specific S100A7-overexpression bi-transgenic (S100A7-OE) and NSG FVB female mouse models. TetO-mS100a7a15 mice were kindly provided by Dr. Yuspa (NIH). TetO-mS100a7a15 mice [18] were cross-bred with MMTV-rtTA mice to generate doxycycline (Dox) inducible mammary gland-specific S100A7-OE mice. Transgenic littermates were genotyped by PCR as described earlier [5]. Female MMTV-mS100a7a15 mice were fed with Dox-chow 1 g/kg (Bio-Serv), and mice with a normal diet served as controls. Mice were anesthetized using isoflurane, and 5X10^5^ murine TNBC cells (MVT1) suspended in PBS were injected into the mammary fat pad of the 4th mammary gland. After the onset of palpable tumors, mice were treated with both inhibitors alone or in combination for up to 4 weeks. As palpable tumors were established, mice were divided into 4 groups: **a.** vehicle control (VC), **b.** Stat3 inhibitor (5 mg/kg; thrice/week; i.p.), **c.** RAGE inhibitor (1 mg/kg twice/week; i.p.) and **d.** combination of Stat3 + RAGE inhibitors (twice/week; i.p.). Tumor size was measured weekly with digital calipers, and volume was calculated according to the formula *V* = 0.52 × *a*^2^ x *b*, where *a* is the smallest superficial diameter and *b* is the largest superficial diameter. Mice were treated for up to four weeks and at the end of the study, mice were euthanized by CO_2_ asphyxiation and cervical dislocation. Tumors and lungs were harvested, formalin-fixed, and paraffin-embedded (FFPE) for histopathology and immunofluorescence (IF) studies. Furthermore, six-week-old female NSG mice were purchased from Charles River. Mice were anesthetized using isoflurane and 1X106^5^ 231 vector control and 231-S7OE cells suspended in PBS were injected into the mammary fat pad of the 4th mammary gland as described above. Tumors were allowed to establish for ~ 2 weeks before treatment with VC, Serpin-E1/PAI1 neutralizing antibody (nAb PAI1, 1 mg/kg), a combination of Stat 3 (5 mg/kg) + RAGE inhibitor (1 mg/kg), and Serpin E1/PAI1 nAb; 1 mg/kg) in combination with Stat3 + RAGE inhibitors. Mice were treated with these inhibitors or PAI1 nAb alone or in combination for up to 3–4 weeks twice/week through i.p. Tumor size were measured weekly with digital calipers and volumes were calculated according to the formula *V* = 0.52 × *a*^2^ x *b.* At the end of the study, mice were euthanized by CO_2_ asphyxiation and cervical dislocation. Tumors, livers, and lungs were harvested, and metastasis nodules in lungs and livers were counted as described earlier [5]. These organs were also formalin-fixed and paraffin-embedded for histology and IF studies.

#### Human cytokine array and serpin E1/PAI-1 ELISA

MDA-MB-231 scramble or 231-S7OE, MDA-MB-468 scramble or S100A7 knockdown (S7KD) TNBC cells were seeded in T25 flasks and grown for 24 h. Then these cells were grown in serum-free media for the next 24 h. Conditioned media (CM) from these cultures were collected, and cytokines secreted by these cells were evaluated using a human cytokine profiling assay kit (R&D Systems) as per the manufacturer’s instructions. The CM from the above culture conditions was also evaluated for the levels of Serpin-E1 by using the Serpin-E1 ELISA kit (R&D Systems) as per the manufacturer’s instructions. In addition, we also analyzed Serpin-E1 levels in the CM harvested from 231-S7OE and MDA-MB-468 cells either treated with VC, Stat3 inhibitor, RAGE inhibitor, and their combination as described above. Densitometry analysis was performed using Image J software.

#### Western blot analysis

TNBC cells were seeded in a T-25 flask and grown for 24 h and then these cells were serum starved for 4 h followed by treatment with RAGE Inhibitor, Stat3 Inhibitor, and their combination for the next 24 h as described above. Cells were harvested and proteins were isolated as described earlier [5]. Monocytic/macrophage cell lines (CRL9855) were seeded and incubated with 10 ng/mL of PMA (phorbol 12-myristate 13-acetate) for 48 h for differentiation. Then, these cells were incubated with CM harvested from 231-S7OE and MDA-MB-468 cells (CM collected 24 h after treatment with vehicle control, Stat3 inhibitor, RAGE inhibitor, or their combination) in a ratio of 1:1 with monocyte/macrophage growth media for a further 5 days. CRL9855 cultured in a 231-S7OE CM was also treated with IgG or Serpin-E1 nAb. After the end of incubation, proteins were isolated from these cells, and lysates were run on SDS-PAGE and transferred to the membrane for the visualization of protein expression as described earlier [5].

### Flow cytometric analysis

Freshly prepared single-cell suspensions from breast tumors were incubated with an Fc receptor-blocking reagent before staining with the following fluorochrome-conjugated mouse antibodies: F4/80-FITC (BM8, #123107, BioLegend), CD8-PE (53–6.7, #100707, BioLegend), CD4-PE/CF594 (RM4.5, #562285, BD Biosciences), CD11b-PE/Cy7 (M1/80, #101216, BioLegend), iNOS-BV421 (CXNFT, #404-5920-82, eBioscience), CD44-BV605 (IM7, #563058, BD Biosciences), CD69-BV650 (H1.2F3, #569688, BD Biosciences), GZMB-FITC (NGZB, #11-8898-82, eBioscience), IFNγ-BV421 (XMG1.2, #505829, BioLegend), TNFα-BV785 (MP6-XT22, #506341, BioLegend), MHC II-BV650 (M5/114.15.2, #107641, BioLegend), and CD45-APC/R700 (30-F11, #565478, BD Biosciences). For surface staining, cells were incubated with antibodies in phosphate-buffered saline (0.1 M PBS, pH 7.4) containing 1% fetal bovine serum (FBS) on ice for 30 min. After staining, cells were washed three times and fixed in 1% paraformaldehyde prior to flow cytometric analysis. For intracellular cytokine staining of TNF-α, IFN-γ, and granzyme B, cells were first stimulated for 4 h with a cell stimulation cocktail (Invitrogen). Surface marker staining was performed as described above, followed by intracellular staining using a standard fixation and permeabilization protocol. Stained cells were analyzed on a Cytek Aurora flow cytometer, and data were analyzed using FlowJo software version 10 (Tree Star, Inc.)[5].

### T cells depletion

CD4^+^ or CD8^+^ T cells depletion was performed in tumor‑bearing RAGE + Stat3 inhibitors treated S100A7 overexpression mice by intraperitoneal injection of anti‑CD4 (clone GK1.5, BE0003‑1, Bio X Cell), anti‑CD8 (clone 53‑6.7, BE0004‑1, Bio X Cell), or control IgG antibodies (BE0090, Bio X Cell) twice weekly. Antibodies were diluted in sterile phosphate‑buffered saline (PBS), and each mouse received 100 µg in a final injection volume of 100 µL [19].

### H&E staining of paraffin-embedded liver and lung sections

Liver and lung tissue sections of FFPE tissues were stained with hematoxylin and counterstained with eosin. These sections were observed under a bright-field microscope. Liver metastasis and lung nodules in VC, RAGE inhibitor, Stat3 inhibitor, and combination-treated groups were counted and plotted as described earlier [5].

### Immunofluorescence (IF) of FFPE tumor sections

FFPE tumor sections were deparaffinized and rehydrated followed by antigen retrieval. Tissue sections were permeabilized in permeabilization buffer and blocked-in blocking buffer as per the manufacturer’s protocol. For Ki-67 staining, tumor sections were incubated with mouse Ki-67 primary antibody overnight at 4^ͦ^C followed by anti-mouse Alexa fluor 488 secondary antibodies. One drop of anti-fade mounting medium containing DAPI was applied to the tumor tissue section on the slide and was examined under the microscope as described earlier [5].

#### Dataset, computational, and statistical analyses

For combination index analysis, we employed CompuSyn 1.0 software and calculated the Combination Index as described earlier [20]. Serpin-E1 expression was analyzed using various publicly available databases/datasets (GENT2, GEPIA, TNMplot, UALCAN, and Metabric data. Single-cell expression of S100A7 in TNBC tumor tissue was analyzed using the Single Cell Portal-Broad Institute database. The correlation of Serpin-E1 with S100A7 and M2-macrophages was determined using the cBioPortal for Cancer Genomics and TIMER2.0 databases. The KM plotter database was used to evaluate the effect of S100A7, Serpin-E1, iNOS (NOS2), and MHCII (HLA-DRB) expressions on OS and RFS in TNBC subtypes. Statistical analysis was performed using appropriate statistical methods (Student t-test, one- and two-way ANOVA) on GraphPad Prism 10. The results are shown as the mean with error bars depicting ± SEM. For continuous variables, two-sample t-tests were used if two groups were compared, and ANOVAs were used if more than 2 groups were compared. *P* < 0.05 was statistically significant. For all graphs, * indicates *P* < 0.05; ** indicates *P* < 0.01; *** indicates *P* < 0.001; **** indicates *P* < 0.0001.

## Results

### S100A7/RAGE enhances breast cancer cells' tumorigenicity by promoting Stat3 phosphorylation

**Fig. 1:**
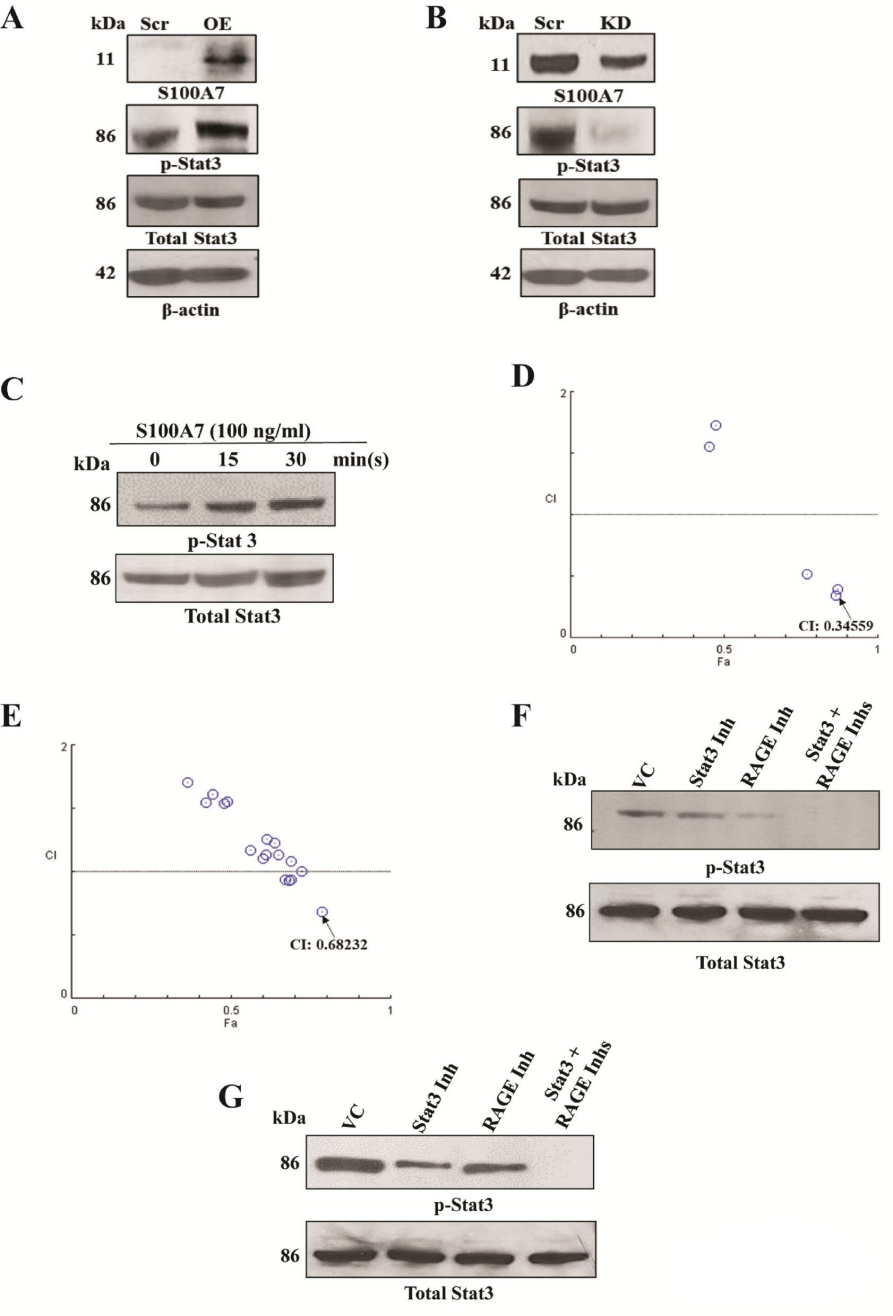
S100A7/RAGE modulates Stat3 phosphorylation in TNBC cells. The cell lysates were harvested from **A** Scramble (Scr) control and S100A7 overexpressing (OE) MDA-MB-231 cells, and **B** Scramble (Scr) control and S100A7 knockdown (KD) MDA-MB-468 cells were analyzed for the level of S100A7, phosphorylation of activated phospho-Stat3 (Ser727) and total Stat3. **C** MDA-M-231 cells were treated with 100 ng of human recombinant S100A7 at different time points and were analyzed for the phosphorylation of activated phospho-Stat3 (Ser727) and total Stat3 using Western blotting. Combination index analysis of **D** S100A7 overexpressing MDA-MB-231 cells, and **E** S100A7 expressing MDA-MB-468 cells treated with different combinations (μM) of Stat3 and RAGE inhibitors. Data are mean ± SEM. (n = 3). Effect of alone or combinatorial treatment of Stat3 and RAGE inhibitors (showing maximum combinatorial synergistic effects) on Stat3 phosphorylation in **F** S100A7 overexpressing MDA-MB-231 cells, and **G** S100A7-expressing MDA-MB-468 cells

S100A7 has been shown to bind to the RAGE receptor [4], whereas Stat3 has been shown to increase S100A7 expression by directly binding to its promoter in breast cancer cells [9]. However, S100A7/RAGE-mediated direct activation of Stat3 and whether a combined inhibition of RAGE and Stat3 suppresses S100A7-mediated breast tumorigenicity have not been characterized. Therefore, here, we analyzed the effect of S100A7/RAGE signaling on Stat3 phosphorylation and thereby its impact on metastatic breast cancer cell's tumorigenicity in-vitro. First, we measured the effect of S100A7 overexpression or its downregulation on Stat3 phosphorylation in metastatic TNBC cells (MDA-MB-231 and MDA-MB-468). We observed that the S100A7 overexpression in MDA-MB-231 cells that does not express S100A7 protein at basal level (S100A7-OE-231) increased Stat3 phosphorylation (Ser727) (Fig. 1A), whereas its downregulation in MDA-MB-468 cells, which express S100A7 at basal level, reduced its activation (Fig. 1B). We also knocked down S100A7 in MDA-MB-468 cells using an independent shRNA clone (clone 2). This knockdown selectively reduced Stat3 phosphorylation at the Ser727 residue, with no notable effect on Tyr705 phosphorylation (Supplementary Fig. 1A), therefore, we focused subsequent analyses on the role of S100A7 signaling in regulating Stat3 Ser727 phosphorylation. Next, we determined the effect of human recombinant S100A7 protein treatment on Stat3 phosphorylation (Ser727) in a time-dependent manner by using MDA-MB-231 cells. Our study revealed that the exogenous treatment of S100A7 on MDA-MB-231 also enhanced the Stat3 phosphorylation in a time-dependent manner (Fig. 1C).

After establishing the effect of S100A7 on Stat3 activation in TNBC cells, we decided to analyze the effect of Stat3 (S3I-201) and RAGE (FPS-ZM1) inhibitors treatment alone or in combination on cell viability of S100A7 expressing (MDA-MB-468) or overexpressing (S100A7-OE-231) TNBC cells at different concentrations for 24 h of time interval. For S100A7-OE-231 cells, Stat3 inhibitor treatment shows an IC_50_ value of ~ 80 μM, (Supplemental Fig. 1B) whereas RAGE inhibitor treatment shows an IC_50_ value of ~ 30 μM (Supplemental Fig. 1C). For MDA-MB-468 cells, the Stat3 inhibitor has an IC_50_ value of ~ 160 μM, (Supplemental Fig. 1D), while RAGE inhibitor treatment has an IC_50_ value of ~ 35 μM (Supplemental Fig. 1E). Next, we performed the combinatorial treatment of Stat3 and RAGE inhibitors using both cell lines at different doses (Supplemental Fig. 1F, G) and analyzed the cell deaths % for the screening of combinatorial doses showing maximal synergism (combination index or CI value < 1.0) using the Chou-Talalay method [20]. After analysis, we found that the combination of 80 μM Stat3 and 50 μM RAGE inhibitors shows maximal synergism with a CI value of 0.345 for S100A7-OE-231 cells (Fig. 1D), while for the MDA-MB-468 cell line, the combination of 160 μM Stat3 and 25 μM RAGE inhibitors shows best synergism with a CI value of 0.682 (Fig. 1E). Lastly, we evaluated the effect of alone IC_50_ and maximal synergistic combinatorial doses of both drugs on the activation of Stat3 by using both S100A7 expressing TNBC cell lines. Here, we observed that the synergistic combinatorial doses of both inhibitors drastically decreased the Stat3 phosphorylation (Ser727) compared to single regimen treatment or vehicle control (VC)-treated cells (Fig. 1F, G).

**Fig. 2:**
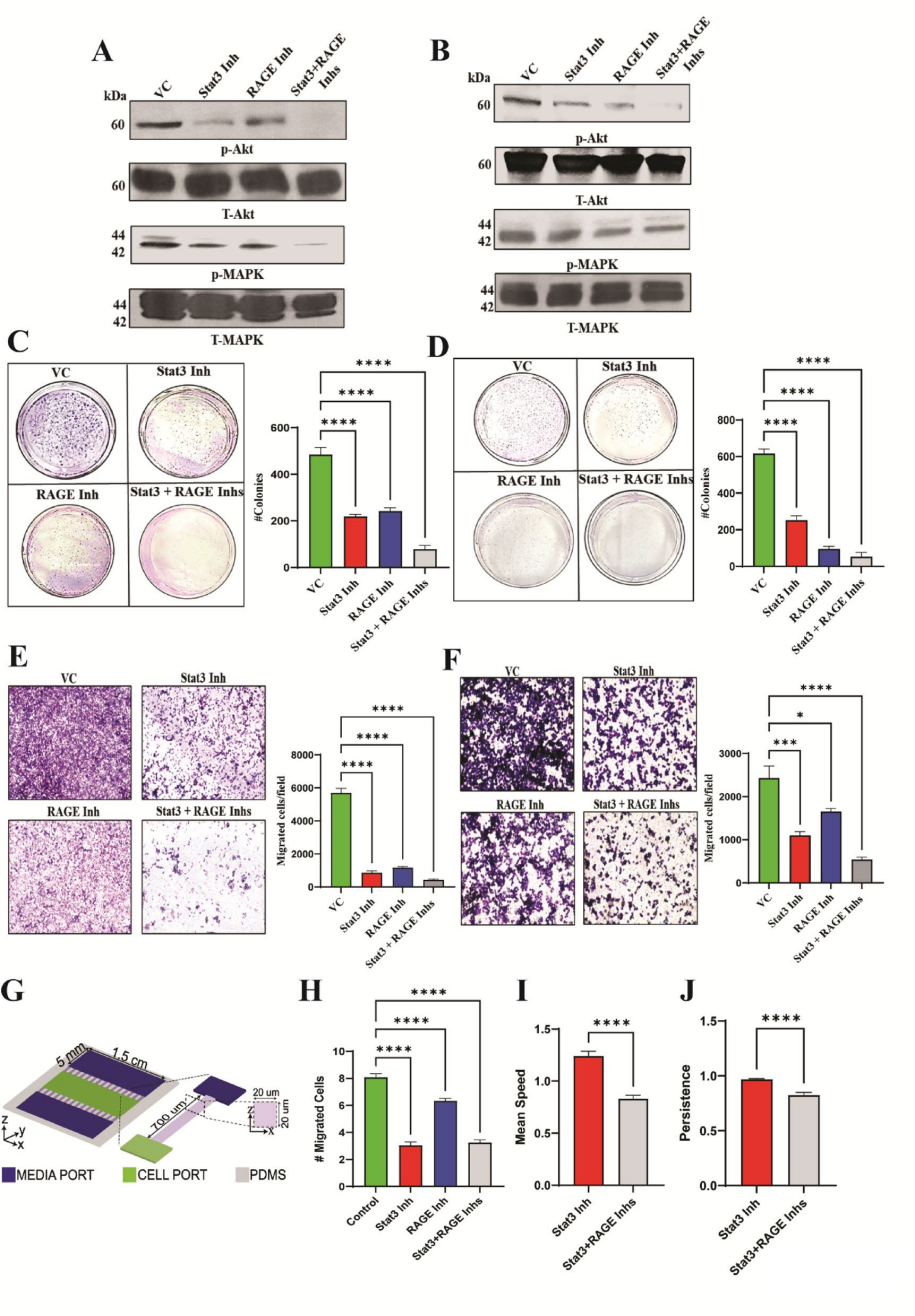
Synergistic effects of RAGE and Stat3 inhibition on S100A7-mediated TNBC tumorigenicity. The cell lysates were harvested from **A** S100A7-expressing MDA-MB-468 cells, **B** S100A7 overexpressing MDA-MB-231 cells treated with vehicle control (VC), IC_50_ values of Stat3 or RAGE inhibitors, and a combination of Stat3 + RAGE inhibitors (showing maximum synergism) and were analyzed for the phosphorylation of activated phospho-Akt (Ser473/474/472) and phospho-MAPK (Thr202/Tyr204) using Western blotting. Total Akt and MAPK were used as loading controls. Effects of Stat3 and RAGE inhibition on colonies forming abilities of **C** S100A7 overexpressing MDA-MB-231 cells, and **D** S100A7-expressing MDA-MB-468 cells. Effects of Stat3 and RAGE inhibition on migration abilities of **E** S100A7 overexpressing MDA-MB-231 cells, and **F** S100A7-expressing MDA-MB-468 cells. **G** Schematic of microfluidic bi-directional microtrack assay. Cells are seeded in the center port (green) and are observed as they migrate through connecting tracks (inset) towards the chemoattractant in the outer ports (blue). Effects of Stat3 or RAGE or combinatorial inhibition on **H** Single cell migration,** I** Mean speed and** J** Persistence of S100A7-OE MDA-MB-231 cells. Data are mean ± SEM. (n = 3). *p < 0.05; ***p < 0.001; ****p < 0.0001

Akt and MAPK signaling cascades are activated in cell proliferation, oncogenesis, and tumor progression in various types of cancer, including breast cancer [21]. Therefore, we also elucidated the effect of individual (IC_50_ doses) and combinatorial treatments of Stat3 and RAGE inhibitors (maximal synergistic doses) on the activation of Akt and MAPK pathways. For both S100A7-expressing TNBC cell lines, the combinatorial treatment of both drugs drastically reduced the phosphorylation of Akt (Ser473/474/472) and MAPK (Thr202/Tyr204) compared to their individual drug treatments or vehicle control-treated groups (Fig. 2A, B). Moreover, we also observed that the alone, as well as the combinatorial treatment of both drugs, significantly reduced the colony-forming abilities of S100A7 expressing MDA-MB-468 and S100A7-OE-231 cells compared to vehicle control-treated cells (Fig. 2C, D). Remarkably, the individual or combination of RAGE and Stat3 inhibitors drastically decreased the migrating and wound-healing abilities of these S100A7-expressing TNBC cells relative to the vehicle control groups (Fig. 2E, F; Supplemental Fig. 2A, B). To exclude the possibility that reduced cell migration resulted from inhibitor-induced cytotoxicity rather than impaired migratory capacity, we performed a single-cell migration assay using a microfluidic device (Fig. 2G) published in our earlier study [18]. S100A7-OE-231 cells were treated with IC₅₀ doses of RAGE inhibitor, Stat3 inhibitor, or their combination, as determined by combination index analysis. Following treatment, equal numbers of viable cells from each group were subjected to single-cell migration analysis. Both individual and combinatorial inhibition of RAGE and Stat3 significantly decreased the number of migrated viable single cells (Fig. 2H). However, no significant difference in the total number of migrated cells was observed between the Stat3 inhibitor alone and the combination treatment groups (Fig. 2H). Notably, quantitative analysis of migratory dynamics revealed that combinatorial treatment led to a significantly greater reduction in mean migration speed and directional persistence compared with Stat3 inhibition alone (Fig. 2I, J), indicating that dual RAGE/Stat3 blockade more effectively impairs intrinsic migratory behavior independent of effects on cell viability. In brief, these findings revealed that S100A7 promotes Stat3 activation and that combinatorial synergistic inhibition of RAGE/Stat3 signaling drastically suppresses S100A7-mediated TNBC cell growth, survival, and migration.

### Combinatorial inhibition of RAGE/Stat3 abrogates S100A7-mediated TNBC burden by enhancing the abundance of anti-tumor MHCII^high^ and iNOS^+^ macrophages

**Fig. 3:**
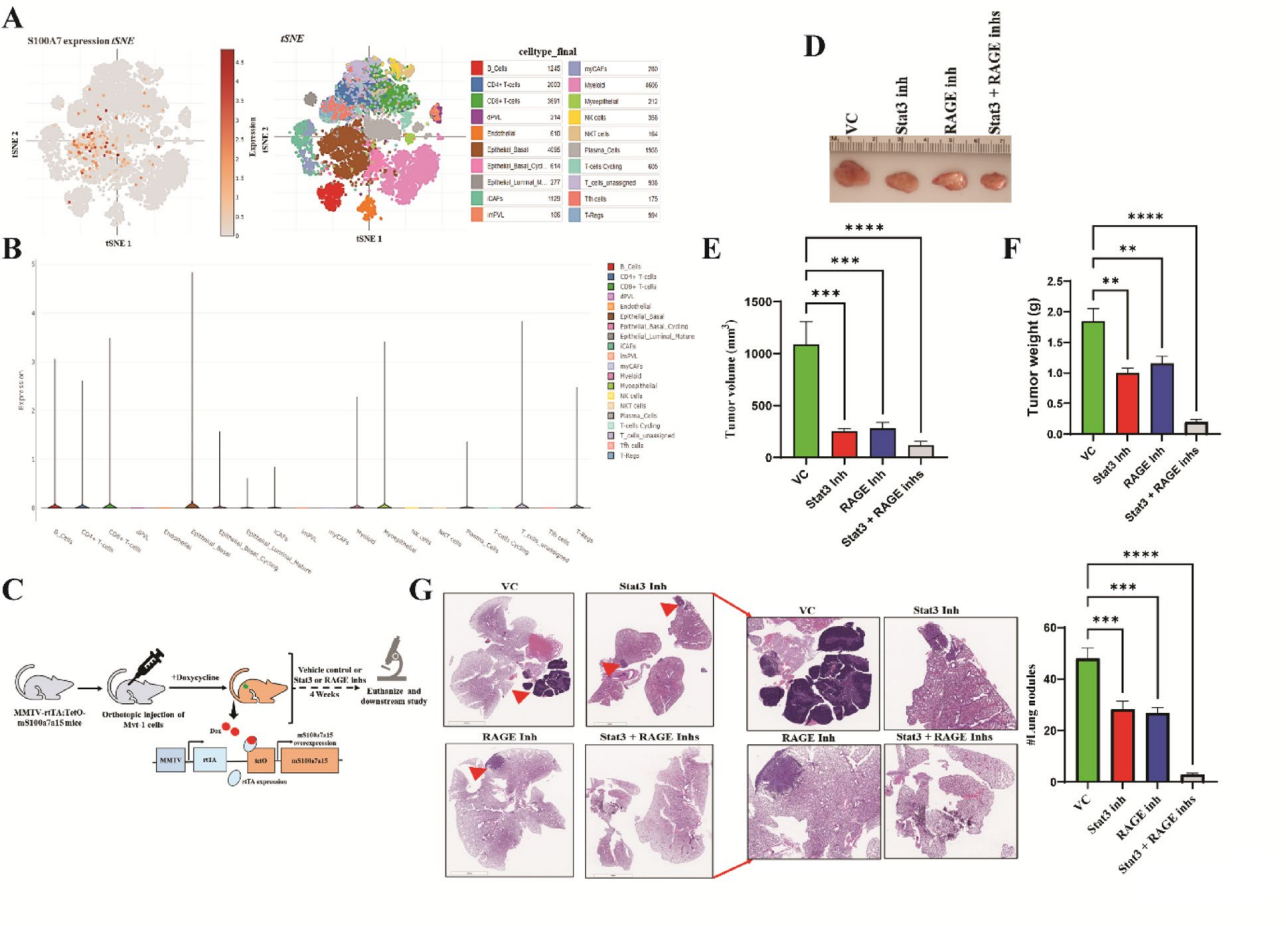
Combinatorial inhibition of Stat3 and RAGE suppresses S100A7-mediated breast tumor burden by modulating the tumor immune microenvironment. **A** t-SNE and **B** Violin plots showing the single-cell expression of S100A7 in various cell types of human TNBC tumor tissues. **C** Schematic diagram of MVT1 cell injection in doxycycline-inducible (+ DOX) S100A7 overexpression bi-transgenic mice and procedure for the downstream experiment. MVT1 cells were injected in the 4th mammary fat pad of S100A7 overexpressing female mice. After the onset of palpable tumors, these mice were treated with vehicle control (VC), Stat3, or RAGE inhibitors alone or their combination for 4 weeks. After treatment, tumor and lung samples were harvested for downstream analysis. N = 5–7 animals** D** Photomicrographs showing the harvested representative tumors and bar diagrams showing the **E** Tumor volume, and **F** Tumor weight of the above-treated groups. **G** The microscopic and Zoom images show the H/E staining of lung nodules in the above-treated mice (left side). The bar diagram shows the number of nodules in each lung (right side). Data are mean ± SEM. (n = 5 to 7). *p < 0.05; **p < 0.01; ***p < 0.001; ****p < 0.0001; ns: non-significant

Previously, we have shown that S100A7 overexpression enhances breast tumorigenesis in-vivo [4, 5]. Moreover, our analysis of a single-cell RNA-seq (scRNA-seq) study of human TNBC tumors using a publicly available dataset revealed that mammary epithelial cells predominantly express S100A7 as compared to other cell types (Fig. 3A, B). Considering this observation, we utilized our doxycycline (DOX)-inducible S100A7 overexpressing bitransgenic mouse model that overexpresses murine mS100a7a15 (an ortholog of human S100A7) predominantly in their mammary epithelial cells [5, 22]. Since we have found that S100A7/RAGE activates Stat3 in TNBC cells, here we analyzed the alone as well as the combinatorial effects of RAGE and Stat3 inhibitors treatment in suppressing aggressive TNBC growth and metastasis using our DOX-inducible S100A7 overexpressing bitransgenic mouse model. In brief, we injected murine metastatic TNBC Mvt1 cell line into the mammary fat pad of these mice provided with DOX in their drinking water and after the onset of palpable mammary tumors, we treated them alone or a combination of RAGE and Stat3 inhibitors for two times a week up to four weeks intraperitoneally (Fig. 3C). After the treatment, we measured the tumor weight and volume harvested from these mice and we discovered that alone treatment of both inhibitors reduced the tumor burden, however, the combinatorial treatment revealed more pronounced and drastic effects on tumor burden compared to the vehicle-treated group (Fig. 3D–F). Next, we also evaluated the effect of these inhibitors on lung metastasis in these mice, as lung metastasis significantly diminishes the survival rates of breast cancer patients [23]. Although the single regimen treatment of these inhibitors reduced lung metastasis in orthotopic tumor-bearing S100A7-overexpressing mice, the combinatorial treatment was more significant in inhibiting lung metastasis of breast cancer cells (Fig. 3G). Increased expression of Ki67 amplifies the breast tumor burden in mice [24]. Therefore, we also determined the expression of Ki67 in tumor tissues harvested from these mice and found that either alone or combinatorial treatment of both inhibitors reduced the expression of Ki67 in tumor tissues (Supplemental Fig. 2C).

**Fig. 4:**
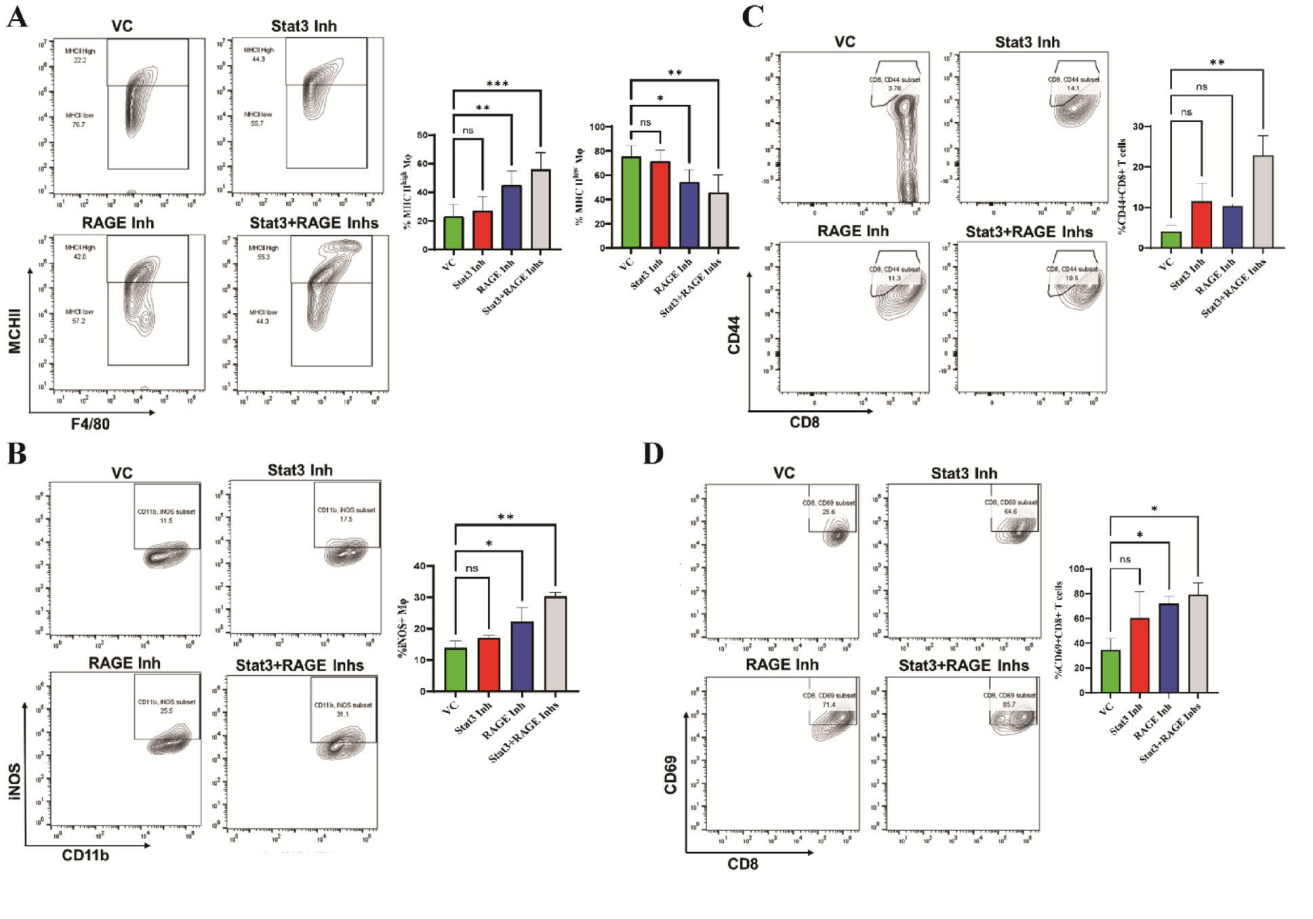
RAGE/Stat3 inhibition reduces S100A7-driven TNBC by promoting MHCII^high^ and iNOS^+^ macrophages and enhancing T-cell-mediated antitumor immunity. The tumors harvested from S100A7-overexpression mice treated with vehicle control, RAGE or Stat3 inhibitors or their combination were analyzed for the different immune cells using multi-color flow cytometry. Effects of alone or combined Stat3 and RAGE inhibition on abundance or infiltration of **A** MHC-II^low^ or MHC-II^high^ **B** iNOS^+^ macrophages, and **C, D** tumor-infiltrating CD44^+^ and CD69^+^ CD8^+^ T lymphocytes. Data are mean ± SEM. (n = 3 to 6). *p < 0.05; **p < 0.01; ***p < 0.001; ****p < 0.0001; ns: non-significant

Understanding the intricate interplay between the immune system and TME is crucial in devising effective therapeutic strategies for breast cancer [25]. Research has highlighted the pivotal role of the tumor immune microenvironment (TIME) in dictating therapeutic response, influencing tumor progression, and impacting patient outcomes [26, 27]. Although our in-vitro studies demonstrated potent cytotoxic effects of RAGE and Stat3 inhibitors against S100A7-expressing TNBC cells, we were also interested in determining whether these inhibitors, alone or in combination, enhance anti-tumor immune responses by modulating the TIME. Importantly, our previous studies have shown that S100A7 overexpression enhances breast tumorigenesis in-vivo by recruiting the immunosuppressive M2-tumor-associated macrophages (TAMs) and depletion of TAMs abrogates the S100A7-mediated tumorigenicity [4, 5], however, whether suppression of S100A7-mediated downstream signaling can attenuate S100A7-induced breast tumor growth by promoting the enrichment of anti-tumor macrophages remains unknown. Therefore, leveraging multi-color flow cytometry, we meticulously assessed the effect of inhibiting the RAGE/Stat3 signaling in S100A7-overexpressing tumors on the composition of anti-tumor macrophages within the TME. After analysis, we observed that the alone or combinatorial treatments of RAGE and Stat3 inhibitors significantly increased the abundance of MHCII^high^ TAMs compared to the vehicle-treated group in tumor bearing S100A7-OE mice model, indicating a shift from a predominantly MHCII^low^ pro-tumorigenic phenotype toward an antigen-presenting, anti-tumorigenic MHCII^high^ macrophage state (Fig. 4A). However, we did not observe any significant change in total abundance of macrophages (Supplemental Fig. 2D). Importantly, a recent study on the effect of combinatorial treatments of anti-cancer drugs in TNBC also identified the reprogramming of MHCII^low^ to MHCII^high^ phenotype in TAMs using different pre-clinical mice models [28].

Classically activated, iNOS-expressing (iNOS⁺) macrophages are widely recognized for their anti-tumor functions, as they produce nitric oxide and pro-inflammatory cytokines that enhance antigen presentation, promote cytotoxic T-cell activity, and limit tumor growth [29–31]. In contrast, alternatively activated macrophages typically display a pro-tumorigenic, immunosuppressive phenotype [29–31]. Therefore, therapeutic strategies that reprogram TAMs toward an iNOS⁺, inflammatory state, are also considered promising approaches to restore anti-tumor immunity. Notably, we observed a marked and statistically significant enrichment of iNOS⁺ anti-tumor macrophages specifically in S100A7-OE tumors treated with the RAGE inhibitor alone or the combination of RAGE and Stat3 inhibitors, compared with vehicle controls (Fig. 4B). This selective expansion of iNOS⁺ macrophages suggests that RAGE blockades play a dominant role in promoting inflammatory macrophage polarization, which is further supported, but not independently driven by Stat3 inhibition. In summary, these data demonstrate that inhibition of RAGE/Stat3 signaling reshapes the TIME by reprogramming TAMs toward an MHCII^high^ and iNOS⁺ anti-tumor phenotype. This macrophage-driven immune reprogramming provides a mechanistic basis for the enhanced anti-tumor immune response observed with RAGE-targeted and combinatorial therapeutic strategies in S100A7-driven metastatic breast cancer.

### Targeting the S100A7/RAGE/Stat3 axis restores CD8⁺ T-cell-dependent immunity and reduces TNBC burden

TNBC is an aggressive malignancy with limited therapeutic options and a profoundly immunosuppressive TIME [32]. Although TNBC is often considered immunogenic, effective anti-tumor immunity is frequently compromised by chronic inflammatory signaling and dysfunctional immune cell programming within tumors [33]. Tumor-resident cytotoxic T lymphocytes (CTL), particularly CD8⁺ effector T cells, are critical mediators of durable anti-tumor responses and are strongly associated with improved patient outcomes and responsiveness to immunotherapy [34]. However, oncogenic inflammatory pathways such as those driven by S100A7 may restrict CTL infiltration, survival, and effector function, thereby promoting tumor progression and distant metastasis.

Our findings show that inhibition of RAGE/Stat3 signaling markedly enriches effector CD8⁺ TILs within S100A7-overexpressing TNBC tumors and enhances their activation status. Flow cytometric analysis revealed significant increases in CD8⁺ T cells expressing activation markers CD44 and CD69, as well as cytotoxic mediators including Granzyme B, and IFNγ without activating TNFα following combined RAGE and Stat3 inhibition (Fig. 4C, D; Supplemental Fig. 3A). CD4⁺ T cells also exhibited enhanced activation and cytotoxic potential, with increased CD44 as well as CD69 expressions and Granzyme B production in alone as well as combinatorial treatments (Supplemental Fig. 3B), however we didn’t observe any significant enrichment of IFNγ, and TNFα expressing CD4^+^ T cells (Supplemental Fig. 4A), suggesting a supportive role in shaping anti-tumor immunity.

**Fig. 5:**
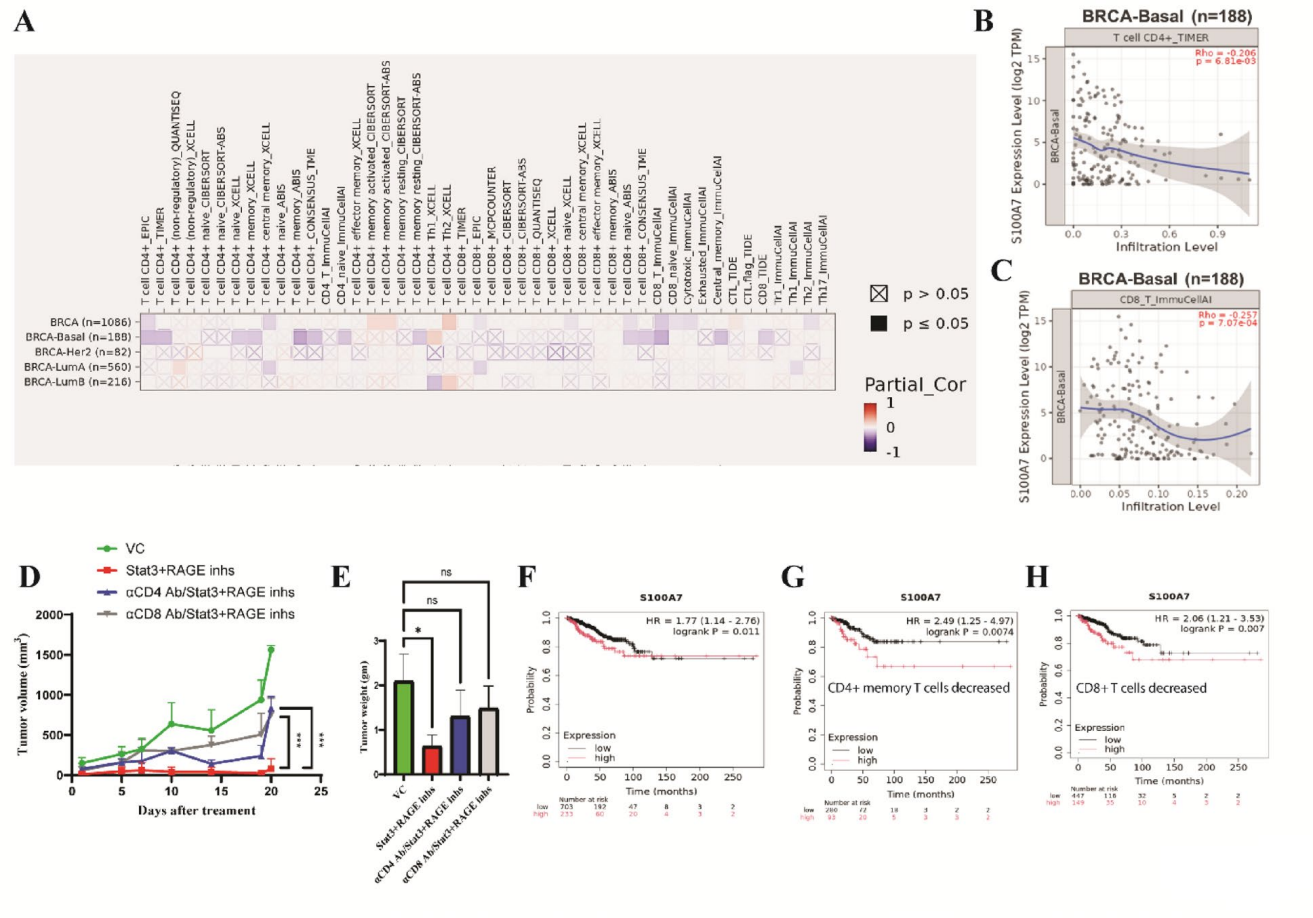
S100A7 limits T-cell infiltration and impairs T-cell-dependent tumor control in TNBC.** A–C** Correlation of S100A7 expression with T-cells infiltration were analyzed in breast cancer subtypes, including the aggressive basal subtype, using the TIMER2.0 database.** D, E** Effect of antibody-mediated depletion of CD8⁺ or CD4⁺ T cells in S100A7-overexpressing mice treated with a combination of RAGE/Stat3 inhibitors. Data are mean ± SEM. (n = 3 to 6). **F, H** Kaplan–Meier analyses reveal that high S100A7 expression predicts poor survival, which is further exacerbated in tumors with low CD4⁺ memory or CD8⁺ T-cell infiltration. *p < 0.05; **p < 0.01; ***p < 0.001; ****p < 0.0001; ns: non-significant

Next, to assess the clinical relevance of S100A7 in shaping TILs-mediated anti-tumor immunity, we analyzed the relationship between S100A7 expression and T-cell infiltration in human breast cancers, including the invasive basal TNBC subtype, using the TIMER2.0 database [35]. Strikingly, S100A7 expression was significantly negatively correlated with overall T-cell infiltration, with the most pronounced effect observed for CD8⁺ cytotoxic T cells in basal breast cancer, except CD4 T cells Th1 and Th2 subsets (Fig. 5A–C). These findings suggest that high S100A7 levels may contribute to immune evasion by limiting the recruitment or persistence of effector T cells within the tumor microenvironment, highlighting its potential as a predictive biomarker for immunosuppressive S100A7-overexpressing TNBC. To further determine whether these T-cell populations are functionally required for the therapeutic benefit of RAGE and Stat3 inhibitors, antibody-mediated depletion studies were performed in tumor-bearing S100A7-OE mice treated with RAGE/Stat3 inhibitors. Depletion of CD8⁺ T cells strongly abrogated tumor regression induced by RAGE/Stat3 inhibition, indicating that CD8⁺ cytotoxic T cells are critical contributors to tumor control in this setting (Fig. 5D, E; Supplementary Fig. 4B). Depletion of CD4⁺ T cells also resulted in a significant reduction in tumor regression, suggesting that CD4⁺ T cells may contribute to, but are not solely responsible for the therapeutic effects of RAGE/Stat3 inhibition (Fig. 5D & E; Supplementary Fig. 4B).

Finally, Kaplan–Meier survival analyses revealed that high S100A7 expression is associated with significantly poorer survival in patients with basal-like breast cancer (Fig. 5F). Notably, the negative impact of S100A7 on prognosis was further exacerbated in tumors exhibiting reduced infiltration of CD4⁺ memory T cells or CD8⁺ cytotoxic T cells. Specifically, S100A7-high tumors with low CD4⁺ memory or CD8⁺ TILs had higher hazard ratios (~ 2.49 and 2.06, respectively) compared to the overall S100A7-high cohort (HR = 1.77) (Fig. 5G, H). These data indicate that the adverse prognostic effect of S100A7 is strongly linked to the suppression of effector and memory T-cell populations, underscoring its role in promoting an immunosuppressive TME that diminishes anti-tumor immunity. These findings underscore the immunosuppressive role of the S100A7/RAGE/Stat3 axis and its impact on T-cell-mediated tumor immunity. In summary, suppression of the S100A7/RAGE/Stat3 signaling axis reprograms the TIME toward effective T-cell-dependent tumor control. The therapeutic efficacy of RAGE/Stat3 inhibition in S100A7-driven TNBC is critically dependent on cytotoxic CD8⁺ T cells, with supportive contributions from CD4⁺ T cells. Collectively, these results identify this pathway as a key immunosuppressive mechanism and highlight its targeting as a promising strategy to restore durable, immune-driven tumor control in invasive S100A7 overexpressing TNBC.

### S100A7/RAGE/Stat3 signaling modulates macrophage phenotypes by regulating Serpin-E1 expression in metastatic TNBC cells

**Fig. 6:**
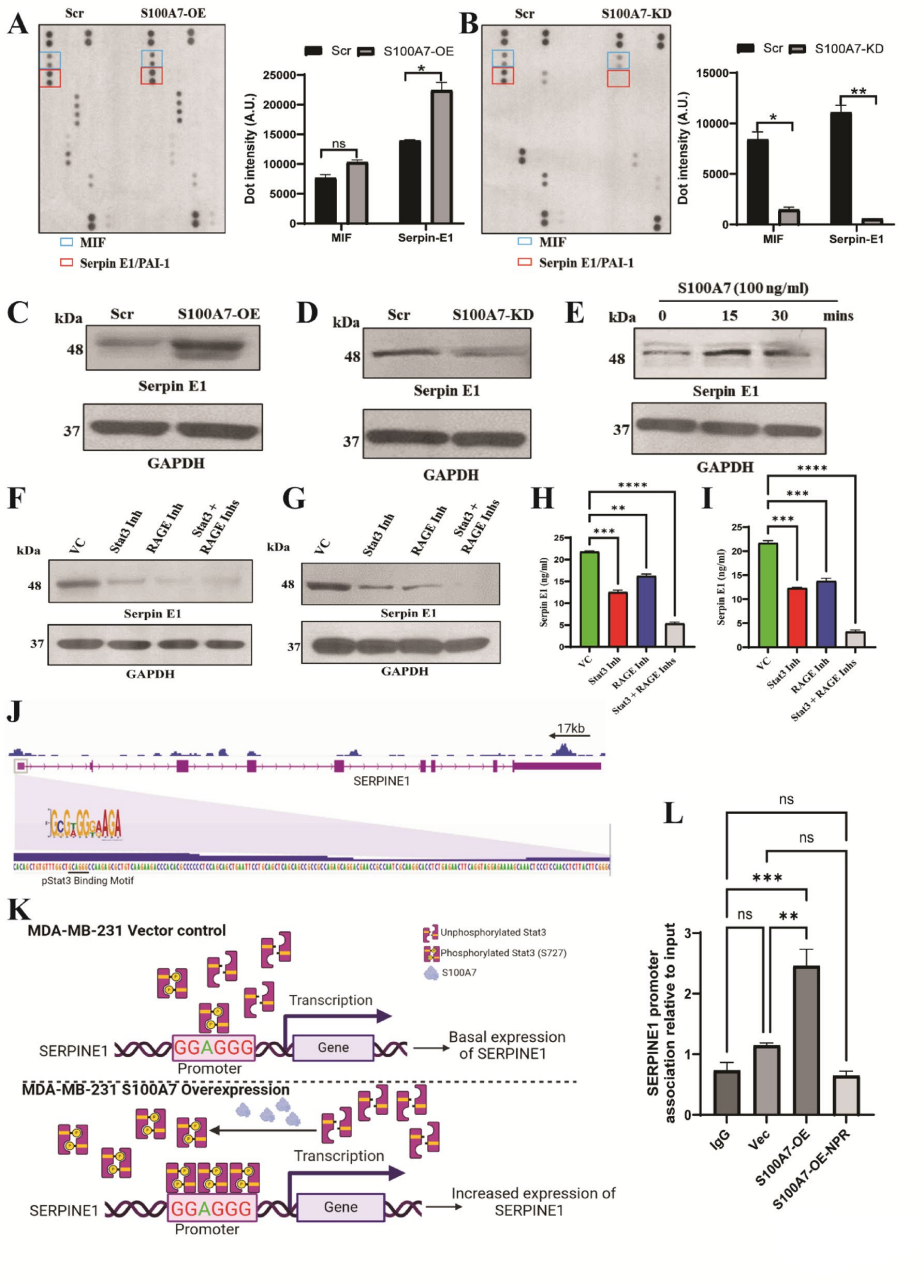
S100A7 modulates the Serpin-E1 (PAI1) expression through RAGE/Stat3 signaling in TNBC cells. Cytokine arrays of conditioned media harvested from **A** Scramble (Scr) control and S100A7-overexpressing (S100A7-OE) MDA-MB-231 cells, **B** Scr and S100A7 knockdown (S100A7-KD) MDA-MB-468 cells. Densitometric analysis of the top two cytokines (MIF and Serpin-E1) regulated by S100A7 in TNBC cells. The cell lysates were harvested from **C** Scramble (Scr) control and S100A7-OE MDA-MB-231 cells, **D** Scr and S100A7-KD MDA-MB-468 cells, and **E** MDA-M-231 cells were treated with 100 ng of recombinant human S100A7 at different time points and were analyzed for the expression of Serpin-E1 using Western blotting. The cell lysates and conditioned media were harvested from **F, H** S100A7-OE MDA-MB-231 cells, and **G, I** S100A7-expressing MDA-MB-468 cells treated with vehicle control (VC), Stat3, or RAGE inhibitors (IC_50_ values) or their combination (showing maximum synergistic effects) were analyzed for expression of Serpin-E1 by Western blotting and ELISA, respectively. **J** ChIP-Seq peak of pStat3 association in SERPINE1 gene showing pStat3 binding sites in the promoter region. The enlarged view of 136 bp shows the binding site of pStat3. **K** Schematic diagram showing the S100A7-mediated upregulation of Serpin E1 by activating the pStat3 (Ser727) in MDA-MB-231 TNBC cells. **L** MDA-MB-231 vector control (Vec) and S100A7 overexpressing (S100A7-OE) cells, and control IgG or pStat3 (Ser727) antibodies were used to perform ChIP and qRT‐PCR was performed to determine SERPINE1 promoter. Non-promoter regions (NPR) from S100A7 overexpressing cells were also used as a negative control. Data are mean ± SEM. (n = 3 to 4). *p < 0.05; **p < 0.01; ***p < 0.001; ****p < 0.0001; ns: non-significant

Next, to understand the S100A7/RAGE/Stat3-mediated mechanism that regulates the anti-tumor macrophages in TNBC, we first performed the cytokine arrays using the S100A7-overexpressing and downregulated TNBC cell lines. We observed that S100A7 overexpression increases, whereas its downregulation reduces the expression of macrophage migration inhibitory factor (MIF) and Serpin-E1 (PAI-1) in metastatic TNBC cells (Fig. 6A, B). After densitometry analysis, we found that S100A7 overexpression or downregulation significantly regulates Serpin-E1 expression rather than MIF expression in these cells (Fig. 6A, B), therefore we decided to evaluate the potential role of S100A7/RAGE/Stat3 signaling in regulating Serpin-E1 in TNBC cells. Further validation using Western blot also indicated that S100A7 overexpression in MDA-MB-231 cells increased, whereas its downregulation in MDA-MB-468 cell lines reduced the Serpin-E1 expression (Fig. 6C, D). Moreover, the human recombinant S100A7-treated MDA-MB-231 cells also showed increased expression of Serpin-E1 protein (Fig. 6E). Next, we mined the expression of Serpin-E1 in normal breast and tumor tissues using different publicly available datasets and we observed a significantly higher level of Serpin-E1 in breast tumor tissues compared to normal breast tissue samples (Supplemental Fig. 5A–D). Additionally, we also discovered a significantly higher level of Serpin-E1 in lymph node-positive breast cancers relative to normal lymph nodes regardless of different stages (Supplemental Fig. 5E). Importantly, the expression of Serpin-E1 was significantly high in ER-PR- or ER + /PR- hormonal subtypes compared to ER + /PR + breast cancer subtype (Supplemental Fig. 5F). Since we observed that S100A7 positively regulate Serpin-E1 expression in breast cancer cells, therefore we also analyzed the correlation of S100A7 with Serpin-E1 mRNA expressions in breast tumor tissue samples using cBioportal genome [36] and we found that S100A7 significantly positively correlated with Serpin-E1 expression in breast tumor tissues, especially in invasive breast carcinomas (Supplemental Fig. 5G). As we have shown that S100A7 enhances Stat3 phosphorylation and Serpin-E1 expression in breast cancer cells and breast tumor tissues express higher levels of Serpin-E1, we next evaluated the effect of inhibiting RAGE and Stat3 in S100A7 expressing or overexpressing TNBC cells on Serpin-E1 protein expression by Western blot and ELISA. We found that alone and combinatorial treatments of RAGE and Stat3 inhibitors drastically reduced the Serpin-E1 expression and its secretion in condition media harvested from these cells (Fig. 6F–I). To investigate whether S100A7-mediated activation of pStat3 (Ser727) leads to its direct binding to the Serpin-E1 promoter in TNBC cells, we performed chromatin immunoprecipitation (ChIP) to assess pStat3 (Ser727) binding at the Serpin-E1 promoter. Initially, we mined the publicly available Stat3 ChIP-Seq data (HRA001909), and next performed genome alignment with hg38 (star alignment) followed by peak calling to see the enrichment of reads (MACS2 v2.1.2), and then converted data to a bigwig for visualization in IGV software (v2.18.2). Motif search is done with MEME motif finder with the ChIP-Seq FASTA sequence file. After analysis, we observed the ChIP-Seq peak of Stat3 association in the SERPINE1 gene promoter (Fig. 6J). We then conducted ChIP-qRT-PCR analysis of the Serpin-E1 promoter bound to pStat3 (Ser727) in MDA-MB-231 vector control and S100A7-overexpressing (S100A7-OE) TNBC cells (Fig. 6K). Control IgG and non-promoter regions (NPR) from S100A7-overexpressing cells served as negative controls in the experiment. After analysis, our study revealed a significant increase in Serpin-E1 expression bound to pStat3 (Ser727) in S100A7-OE cells compared to vector control cells (Fig. 6L).

**Fig. 7:**
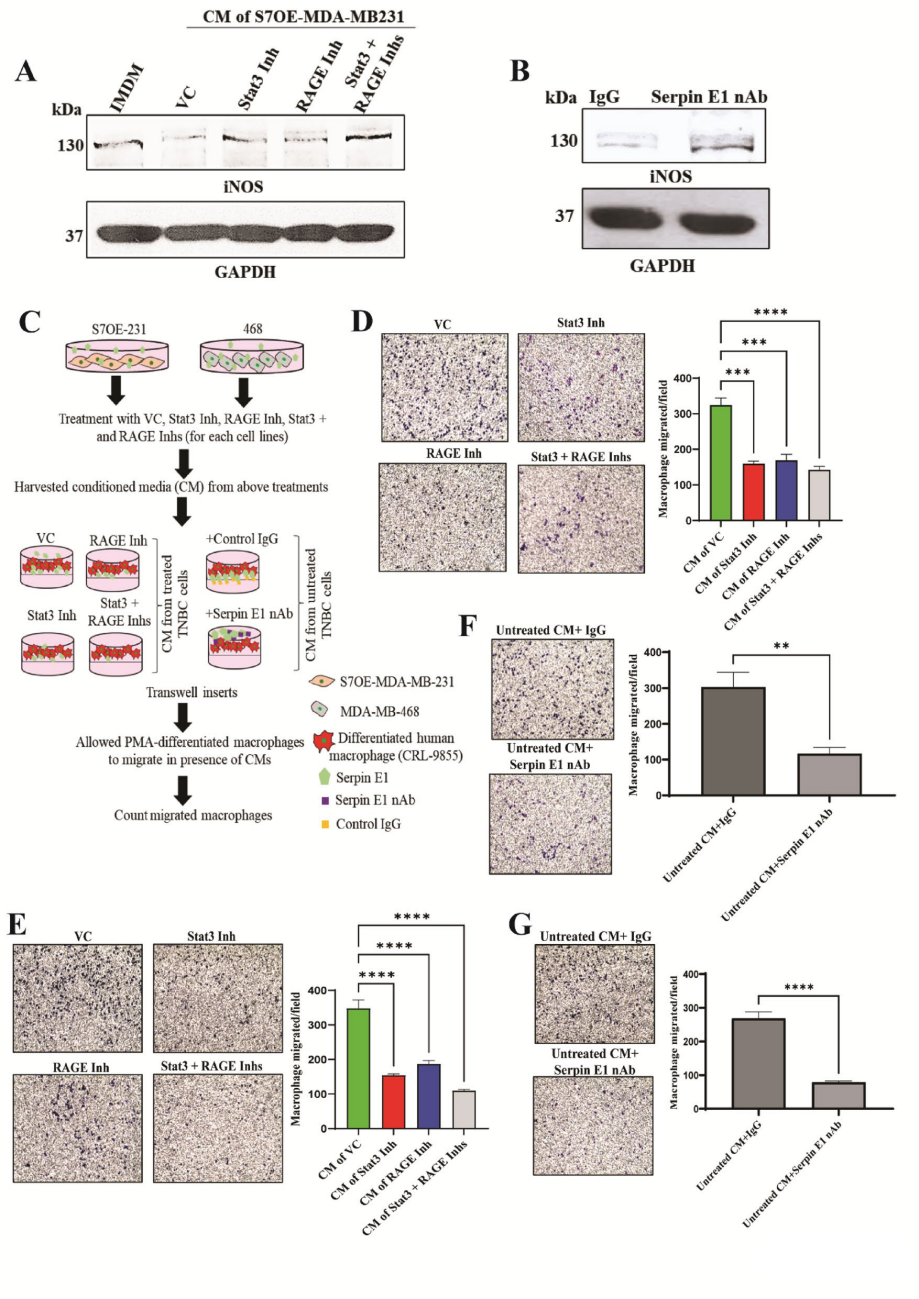
S100A7/RAGE/Stat3-mediated regulation of Serpin-E1 modulates macrophage polarization and migration in TNBC. **A** The cell lysates harvested from human macrophage cells incubated with IMDM (untreated basal medium) or conditioned media (CM) collected from S100A7-overexpressing MDA-MB-231 cells treated either with vehicle control (VC) or, Stat3 or RAGE inhibitors alone or in combination (CM collected 24 h after treatment with vehicle control, STAT3 inhibitor, RAGE inhibitor, or their combination to avoid any direct toxicity on macrophage) and were analyzed for the expression of anti-tumor MHC-II^high^ macrophage marker (iNOS) by Western blotting. **B** The cell lysates harvested from human macrophage cells incubated with conditioned media collected from S100A7-overexpressing MDA-MB-231 cells, with the treatment of control IgG or Serpin-E1 nAb and were analyzed for the expression of iNOS by Western blotting. **C** Schematic diagram of experimental strategy for studying the effect of conditioned media harvested from S100A7-overexpressing MDA-MB-231 and S100A7-expressing MDA-MB-468 cells treated either with vehicle control (VC) or Stat3 or RAGE inhibitors alone or in combination on human macrophage cell migration. Additionally, conditioned media collected from untreated S100A7-overexpressing MDA-MB-231 or S100A7-expressing MDA-MB-468 cells were also used to evaluate the effect on macrophage migration in the presence of control IgG or Serpin-E1 neutralizing antibody (nAb). Microscopic images show the migration of human macrophage cells incubated with conditioned media harvested from **D** S100A7-overexpressing MDA-MB-231 cells, and **E** S100A7-expressing MDA-MB-468 cells were treated either with vehicle control (VC) or, Stat3 or RAGE inhibitors alone or in combination, and migration assays were performed using transwell migration assays. Microscopic images show the migration of human macrophage cells incubated with conditioned media harvested from untreated **F** S100A7-overexpressing MDA-MB-231, and **G** S100A7 expressing MDA-MB-468 cells in the presence of control IgG or Serpin-E1 nAb, and migration assays were performed using transwell migration assays. Data are mean ± SEM. (n = 3). **p < 0.01; ***p < 0.001; ****p < 0.0001; ns: non-significant

Serpin-E1, also known as PAI-1, is synthesized by various cell types, including cancer cells, and plays a crucial role in cancer progression [14]. It acts as a pro-tumorigenic factor by facilitating the migration and polarization of macrophages within the TME by activating the Stat3 pathway [14]. Moreover, the elevated expression of Serpin-E1 is associated with a higher abundance of tumor-promoting M2-type TAMs in cancer [14]. Using the TIMER2.0 database [35], we also observed the significantly positive association of Serpin-E1 with the infiltration of M2-TAMs in the aggressive basal type of breast cancer (Supplemental Fig. 5H). Since, RAGE/Stat3 inhibition in tumor-bearing S100A7-OE mice increased MHCII^high^ and iNOS^+^ macrophages in the TME, which exert antitumor effects by producing nitric oxide and reactive nitrogen species to induce tumor cell apoptosis, while also enhancing CD8 T cell recruitment, activation, and tumor infiltration for improved anti-cancer responses [37], therefore we evaluated the effect of inhibiting the S100A7/RAGE/Stat3/Serpin-E1 signaling in enhancing the iNOS expression in human macrophages incubated with conditioned media (CM) of RAGE/Stat3 inhibitors treated S100A7-expressing TNBC cells or CM supplemented with Serpin-E1 nAb. Here, we observed that macrophages incubated with CM of S100A7 overexpressing MDA-MB-231 cells treated alone or a combination of RAGE and Stat3 inhibitors (fresh CM collected 1 day post-treatment to rule out any direct cytotoxic effects of the inhibitors on macrophages) revealed a higher level of iNOS compared to macrophages incubated with CM harvested from vehicle control treated S100A7-OE TNBC cells (Fig. 7A). Importantly, we also discovered that Serpin-E1 neutralization increased the iNOS expression in macrophages incubated with CM of S100A7 overexpressing MDA-MB-231 cells (Fig. 7B).

Considering these results, we also decided to elucidate the effects of inhibiting the S100A7/RAGE/Stat3/Serpin-E1 signaling in suppressing macrophage migration exposed to CM of S100A7-expressing TNBC cells using RAGE/Stat3 pharmacological inhibitors or Serpin-E1 neutralizing antibody (nAb) (Fig. 7C). In this study, we treated the S100A7 expressing TNBC cells with an alone or combinatorial dose of RAGE and Stat3 inhibitors and then after the completion of treatment, we added the fresh media and collected the CM from these treated cells 1 day after post-treatment. Next, the PMA-differentiated human macrophages were allowed to migrate either in the presence or absence of these collected CM overnight. We also collected the CM from these untreated S100A7-overexpressing MDA-MB-231 TNBC cells and incubated the macrophages with control IgG or Serpin-E1 nAb. We observed that the macrophages incubated with CM harvested from S100A7-expressing TNBC cells treated alone, as well as both inhibitors of Stat3 and RAGE revealed significantly reduced migration compared to conditioned media of vehicle-treated cells (Fig. 7D, E). Similar observations were noticed in Serpin-E1 nAb-treated macrophages compared to the control IgG-treated group (Fig. 7F, G), indicating that S100A7-driven signaling regulates both macrophage polarization and migratory capacity. The reduced migration observed in the treatment group likely reflects a decrease in MHCII^low^ immunosuppressive TAMs, consistent with our in-vivo observations. Notably, the migrated macrophages that remain are iNOS^+^ MHCII^high^, indicative of a pro-inflammatory, anti-tumor phenotype. These findings suggest that the treatment selectively limits immunosuppressive TAMs recruitment while preserving or promoting the migration of anti-tumor macrophages. Altogether, our results indicate that S100A7 enhances Serpin-E1 expression in TNBC cells through the RAGE/Stat3 axis. Furthermore, the combinatorial inhibition of RAGE/Stat3 or Serpin-E1 neutralization decreased the S100A7/Serpin-E1-mediated macrophage migration and increased the expression of the iNOS marker associated with the MHCII^high^ TAMs phenotype.

### Serpin‑E1 neutralization enhances the antitumor and anti‑metastatic activity of RAGE/Stat3 inhibitors in S100A7‑expressing TNBC tumors

**Fig. 8:**
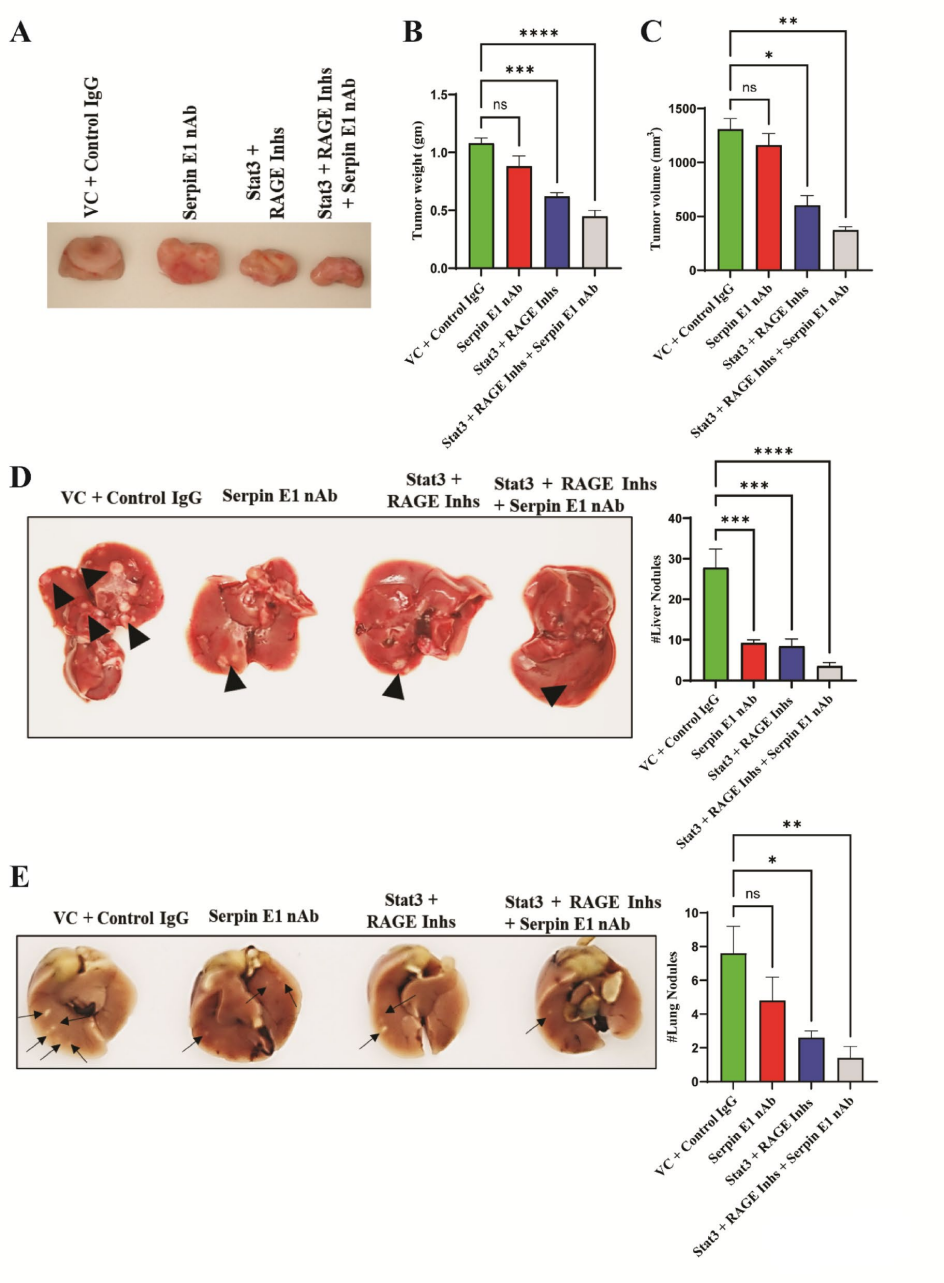
Combined synergistic effect of Serpin-E1 nAb with Stat3/RAGE inhibitors on S100A7-mediated TNBC growth and metastasis. S100A7 overexpressing MDA-MB-231 cells were injected into the 4th mammary fat pad of 6-week-old female NSG mice. After the onset of palpable tumors, mice were treated with vehicle control (VC) + control IgG, Serpin-E1 nAb, the combination of Stat3 + RAGE inhibitors, or a combination of Stat3 + RAGE inhibitors + Serpin-E1 nAb for 4 weeks and were analyzed for tumor growth and metastasis.** A** Photomicrographs show the harvested representative tumors. **B** Tumor weight, and **C** Tumor volume of the above-treated mice. Photomicrographs and bar diagrams show the **D** Liver nodules, and **E** Lung nodules of the above-treated mice. Data are mean ± SEM. (n = 3 to 5). *p < 0.05; **p < 0.01; ***p < 0.001; ****p < 0.0001; ns: non-significant

Since we demonstrated that S100A7/Stat3 signaling regulates Serpin-E1 expression in TNBC cells (Supplemental Fig. 6A) and modulates the MHCII^high^ and iNOS^+^ macrophage phenotype, we next evaluated the therapeutic relevance of targeting this axis in-vivo using an NSG mouse model. NSG mice lack functional adaptive immune cells and exhibit well-recognized defects in several innate immune compartments. Nevertheless, residual monocyte and macrophage populations are present and can be interrogated in-vivo. Accordingly, this model permits focused assessment of tumor-macrophage interactions and macrophage-associated processes, such as phagocytic activity and anti-tumor functions, under conditions that minimize contributions from adaptive immunity as described in earlier studies [38, 39]. To this end, S100A7-overexpressing MDA-MB-231 cells were orthotopically injected into the mammary glands of 6-week-old female NSG mice. Following the onset of palpable tumors, mice were treated with vehicle control (VC), Serpin-E1 neutralizing antibody (nAb) alone, combined Stat3 + RAGE inhibitors, or the triple combination of SerpinE1 nAb + Stat3 + RAGE inhibitors. The primary objective of this study was to determine whether neutralization of Serpin-E1 enhances the therapeutic efficacy of Stat3 + RAGE inhibition in an S100A7-dependent tumor model. We observed that Serpin-E1 nAb alone did not significantly reduce primary tumor burden compared to VC-treated mice. In contrast, treatment with Stat3 + RAGE inhibitors significantly inhibited primary tumor growth, and this effect was maintained or modestly enhanced by the addition of Serpin-E1 nAb (Fig. 8A–C). Analysis of metastatic dissemination revealed that Serpin-E1 neutralization alone significantly reduced liver metastasis, whereas Stat3 + RAGE inhibition, either alone or in combination with Serpin-E1 nAb, robustly suppressed liver metastatic burden (Fig. 8D; Supplemental Fig. 6B). Notably, Stat3 + RAGE inhibition significantly reduced lung metastasis, and the addition of Serpin-E1 nAb further enhanced this therapeutic response (Fig. 8E; Supplemental Fig. 6C). In contrast, Serpin-E1 nAb alone had no significant effect on lung metastatic burden compared to VC-treated mice.

To directly assess whether these therapeutic effects are mediated through S100A7 signaling, we performed parallel in-vivo studies using MDA-MB-231 vector control cells that does not express S100A7 protein and also show very low levels of activated Stat3 or Serpin-E1 expression. Under identical treatment conditions, mice bearing S100A7-low tumors exhibited no significant changes in primary tumor growth or metastatic burden in response to Stat3 + RAGE inhibitors, either alone or in combination with SerpinE1 nAb, compared to VC-treated controls (Supplemental Fig. 6D–F). These findings indicate that the antitumor effects of Stat3 + RAGE inhibition and their enhancement by Serpin-E1 neutralization are largely dependent on S100A7 expression in-vivo. Collectively, these data clarify that although S100A7, Stat3, and Serpin-E1 are components of a linear signaling axis, and combinatorial therapeutic effects observed in-vivo likely reflect context-dependent contributions of downstream effectors within the macrophage-containing TME. Importantly, the absence of therapeutic responses in S100A7-negative tumors establishes that these effects are mediated primarily through S100A7-driven signaling rather than S100A7-independent pathways.

### S100A7/SERPINE1 co-expression identifies a poor-prognosis TNBC subset modulated by macrophage activation

**Fig. 9:**
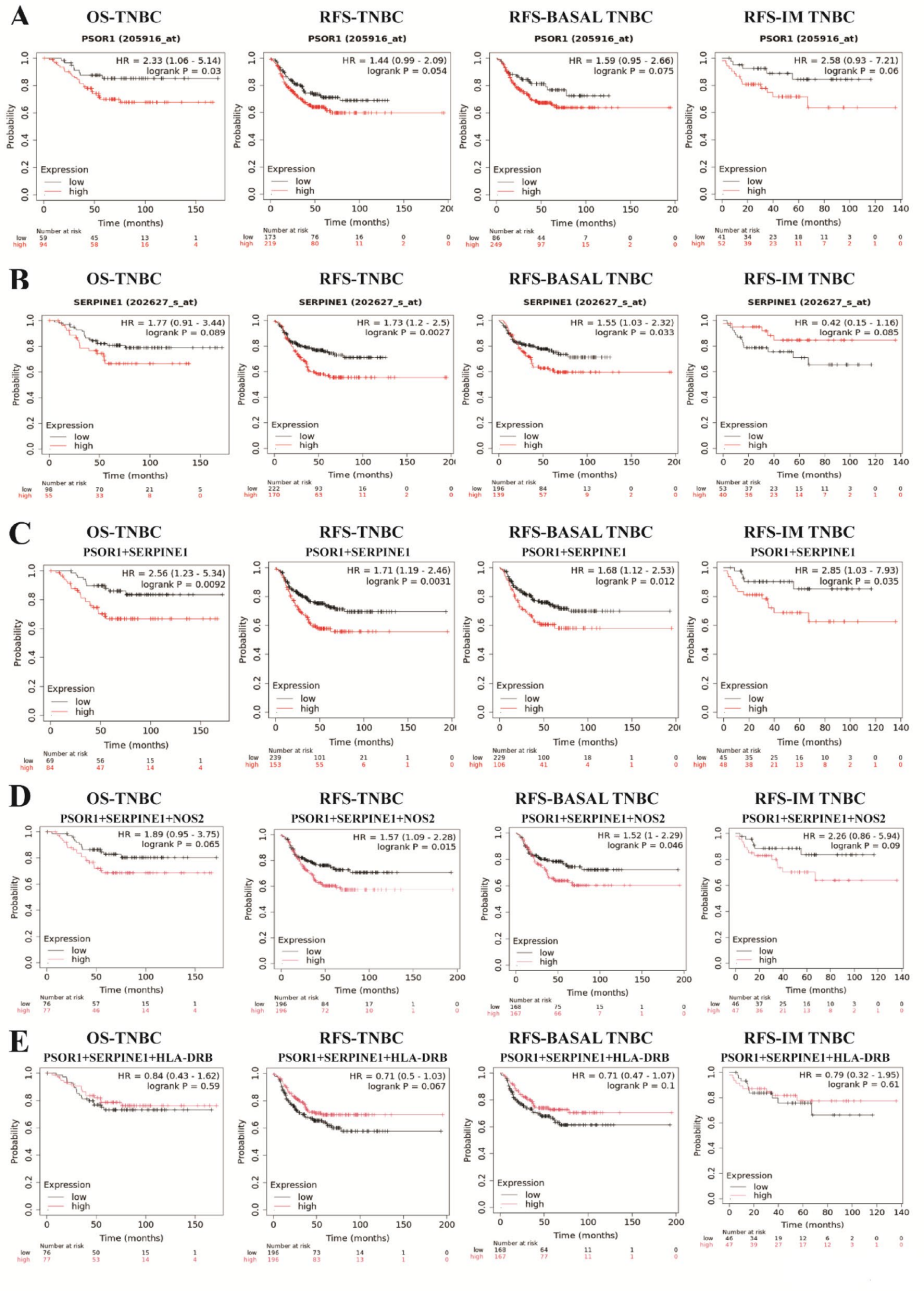
Combined S100A7 and SERPINE1 expression predicts poor survival in TNBC and is modulated by macrophage-associated markers. Kaplan Meier (KM) plot analysis showing the effect of **A** S100A7 (PSOR1), **B** Serpin-E1 (SERPINE1), **C** PSOR1 + SERPINE1, **D** PSOR1 + SERPINE1 + NOS2, and **E** PSOR1 + SERPINE1 + HLA-DRB on overall (OS) and recurrence-free survival (RFS) of TNBC, basal TNBC, and IM TNBC subtypes

Recently, SERPINE1 has been reported as a prognostic and immunological biomarker in pan-cancer [40]. The high expression of Serpin-E1 has been reported in ER- breast cancer and basal B subtype of breast cancer. Moreover, high expression of Serpin-E1 has been found in overweight and obese TNBC patients compared to normal BMI TNBC patients [12]. Importantly, a recent study indicated that high expression of Serpin-E1 is associated with poor overall survival (OS) of TNBC patients [12]. However, the impact of Serpin-E1 with other molecular markers on OS and recurrence-free survival (RFS) in TNBC has not been evaluated. Therefore, in this study, we analyzed the alone as well as combined effects of S100A7 (PSOR1) and SERPINE1 genes on OS and RFS of TNBC patients using the Kaplan–Meier (KM) plotter tool [41]. After analysis, we discovered that combined high expression of PSOR1 and SERPINE1 genes was highly significant (*p* = 0.0092) associated with decreased OS of TNBC patients compared to alone expression of both genes (PSOR1 = 0.03; SERPINE1 = 0.089) (Fig. 9A–C). Furthermore, the high expression of PSOR1 was not significantly associated with reduced RFS of TNBC patients (*p* = 0.054) (Fig. 9A), although the high expression of SERPINE1 and the combination of both genes were significantly equally associated with decreased RFS of TNBC patients (SERPINE1 = 0.0027; PSOR1 + SERPINE1 = 0.0031) (Fig. 9B, C). Basal-like breast cancer shares similarities with TNBC due to the frequent absence of estrogen, progesterone, and HER2 receptors in tumor cells, and gene expression profiling often identifies TNBC as a subtype of basal-like breast cancer [42]. Thus, we also analyzed the effects of alone and combined high expression of both genes on RFS of the basal subtype of TNBC and we discovered that the combined high expression of both genes (PSOR1 + SERPINE1 = 0.012) was more significantly associated with reduced RFS of basal subtype of TNBC patients as compared to alone expression of PSOR1 (*p* = 0.075) and SERPINE1 (*p* = 0.033) (Fig. 9A–C). The immunomodulatory (IM) subtype of TNBC is characterized by high immune infiltration and good prognosis [43]. Since we found that S100A7/Serpin-E1 signaling regulates TIME by modulating MHCII^high/low^ or iNOS^+^ macrophages and TILs, we also determined the effect of high expression of both genes on RFS of the IM subtype of TNBC. Notably, the combined high expression of both genes (PSOR1 + SERPINE1 = 0.035) was significantly associated with reduced RFS in TNBC patients; however, the alone expression of PSOR1 (*p* = 0.06) and SERPINE1 (*p* = 0.085) was not significantly associated with RFS in the IM subtype of TNBC (Fig. 9A–C).

In our in-vivo studies, inhibition of S100A7 signaling was associated with a marked increase in the abundance of MHCII^high^ and iNOS^+^ macrophage populations. Based on these observations, we next evaluated whether incorporating macrophage-associated markers, iNOS (NOS2) and MHC class II (HLA-DRB), modified the prognostic impact of PSOR1 and SERPINE1 expression on OS and RFS in aggressive TNBC subtypes. Inclusion of NOS2 in the survival models substantially reduced the hazard ratios (HRs) across all TNBC subtypes (Fig. 9D). Notably, NOS2 inclusion mitigated the adverse prognostic effect associated with high PSOR1 and SERPINE1 expression, abrogating the poor OS observed in TNBC patients and the reduced RFS observed in IM-TNBC patients (Fig. 9D). We further assessed the impact of incorporating HLA-DRB together with S100A7 and SERPINE1 on OS and RFS. Inclusion of HLA-DRB markedly reduced both the HRs and the statistical significance of the poor survival outcomes associated with high S100A7 and SERPINE1 expression (Fig. 9E). Although HLA-DRB inclusion did not independently confer a statistically significant improvement in OS or RFS, it consistently attenuated the overall adverse prognostic effect in TNBC patients exhibiting high PSOR1 and SERPINE1 expression (Fig. 9E). Altogether, these findings demonstrate that combined high expression of PSOR1 and SERPINE1 is associated with significantly reduced OS and RFS in TNBC patients, with particularly pronounced effects in basal-like and IM subtypes. Importantly, incorporation of anti-tumor macrophage-associated immune markers, such as NOS2 and HLA-DRB, markedly attenuated the adverse prognostic impact of PSOR1 and SERPINE1, suggesting that anti-tumor macrophage activation status may modulate the clinical outcomes of these aggressive TNBC subsets. These results highlight a potential protective role for pro-inflammatory and antigen-presenting macrophage phenotypes in counteracting PSOR1/SERPINE1-driven tumor aggressiveness. Collectively, our data supports further investigation of therapeutic strategies that simultaneously target PSOR1/SERPINE1 signaling while promoting anti-tumor macrophage activation to improve outcomes in TNBC patients.

## Discussion

Metastatic breast cancer, particularly TNBC, remains a formidable challenge in clinical oncology due to its aggressive behavior and the limited efficacy of available treatment options [44]. In this study, we demonstrate that S100A7 activates Stat3 through RAGE-dependent phosphorylation at Ser727, but not Tyr705, in TNBC cells, and that combinatorial inhibition of RAGE and Stat3 synergistically suppresses the viability and tumorigenic potential of S100A7-expressing TNBC cells in-vitro. We revealed that the combined pharmacological blockade of RAGE and Stat3 reduced the activation of cancer cell survival markers (Akt and MAPK) in S100A7-expressing TNBC cells. Importantly, we revealed that either alone or combinatorial treatment of RAGE and Stat3 inhibitors significantly reduced the colony forming and invasiveness of S100A7-expressing TNBC cells in-vitro. Interestingly, combinatorial inhibition of RAGE and Stat3 intrinsically impairs S100A7-mediated cell migration by attenuating the mean speed and persistence using single-cell assays, independent of proliferation effects. Recently, it has also been shown that dual inhibition of Akt and MAPK in TNBC cells potentiates the anti-cancer effects of EGFR inhibitors against TNBC [45]. Therefore, the combinatorial treatment of RAGE and Stat3 inhibitors could also be used with EGFR inhibitors to synergistically suppress the progression of aggressive S100A7-overexpressing TNBC.

Our study also provided valuable insights into the complex interplay between S100A7/RAGE/Stat3 signaling and the immune TME within TNBC tumors. By inhibiting both Stat3 and RAGE, we observed a synergistic suppression of aggressive breast tumor growth and lung metastasis in DOX-inducible mammary gland-specific S100A7-OE mice. Importantly, this combined inhibition also enhanced the infiltration of anti-tumor immune cells, such as MHCII^high^/iNOS^+^ anti-tumor macrophages and CD4^+^ as well as CD8^+^ effector and tumor resident T cells, into the TME, suggesting a potential immunomodulatory effect of this treatment strategy. Importantly, antibody-mediated depletion of CD4⁺ and CD8⁺ T cells significantly abolished the RAGE/Stat3 inhibitors conferred tumor regression, confirming their essential role. Moreover, S100A7 expression showed a strong inverse association with overall tumor-infiltrating T cells, and its adverse prognostic impact was further amplified in tumors with reduced infiltration of CD4⁺ memory T cells or CD8⁺ cytotoxic T cells.

M1-like and M2-like TAMs exhibit distinctive traits corresponding to MHCII^high^ and MHCII^low^ TAMs, respectively [46]. Typically, MHCII^high^ TAMs are associated with early-stage tumor development and demonstrate tumor-suppressive functions, whereas MHCII^low^ TAMs become prevalent during progression phases and tend to promote tumor growth [46]. Interestingly, recent studies have shown the capability of CD4^+^ T cells to directly induce tumor cell death in breast tumors expressing MHC-II [47–49]. Notably, CD4^+^ TILs have been found to stimulate the differentiation of monocytes into MHCII^high^ TAMs with anti-tumor properties [50]. In summary, the results of our studies indicate that combining RAGE and Stat3 inhibitors effectively suppressed the growth of S100A7-overexpressing TNBC and reduced distant lung metastasis. Additionally, this treatment strategy enhanced the infiltration of anti-tumor immune cells, especially MHCII^high^ TAMs in the TME, offering the potential for augmenting the therapeutic efficacy of FDA-approved immunotherapy drugs against TNBC in future applications.

Our study also investigated the downstream effectors involved in attenuating S100A7-mediated tumor growth and metastasis by RAGE and Stat3 inhibitors, focusing on macrophage recruitment and anti-tumor phenotypes in the TME. We identified Serpin-E1 (PAI-1) as a key molecule involved in these processes. Serpin-E1, classically known for its role in regulating fibrinolysis, was found to promote the recruitment and polarization of pro-tumorigenic macrophages in cancer while also exhibiting an inhibitory effect on tumor cell apoptosis [14]. Elevated expression of Serpin-E1 was associated with activation of tumor and immune-related pathways, as well as poor prognosis in carcinogenesis [40]. Our findings also revealed a positive correlation between Serpin-E1 expression and increased infiltration of pro-tumorigenic M2-TAMs in basal subtypes of invasive breast carcinoma. Analysis of publicly available databases confirmed elevated Serpin-E1 expression in breast tumor tissues, particularly in metastatic, lymph node-positive, and ER-/PR- breast cancer patients, with a positive correlation with S100A7 expression. Moreover, we revealed that suppression of S100A7/RAGE/Stat3 signaling resulted in decreased Serpin-E1 expression in TNBC cells. Moreover, inhibition of RAGE/Stat3 or neutralization of Serpin-E1 inhibited macrophage migration exposed to S100A7-expressing TNBC cells conditioned media and increased the expression of iNOS, a marker for anti-tumor TAMs. Notably, neutralization of Serpin-E1 significantly enhanced the anti-metastatic efficacy of RAGE and Stat3 inhibition in-vivo only in S100A7-expressing TNBC models, supporting its role as a critical downstream effector of this signaling axis. In contrast, inhibition of RAGE, Stat3, or Serpin-E1 did not significantly reduce tumor burden in mice bearing TNBC tumors lacking S100A7 expression, underscoring the context-dependent nature of this pathway. Importantly, the observed reduction in metastatic burden in S100A7-expressing tumors primarily reflects suppression of primary tumor growth, while Serpin-E1 neutralization provided additional anti-metastatic benefit beyond tumor inhibition alone. This finding supports a distinct role for Serpin-E1 in regulating metastatic progression rather than simply reflecting reduced tumor size. Collectively, these data identify Serpin-E1 as a key mediator of S100A7-driven RAGE/Stat3 signaling that shapes macrophage recruitment, polarization, and anti-tumor immune responses within the TME. Thus, co-targeting Serpin-E1 together with RAGE and Stat3 represents a promising therapeutic strategy to enhance anti-tumor immunity and limit metastasis in aggressive, S100A7-positive TNBC.

Our investigation also underscored the significance of considering S100A7 and Serpin-E1 mutually as prognostic and immunological molecular markers in TNBC subtypes. Our study adds to recent research findings that report the high expression of Serpin-E1 in overweight and obese TNBC patients, which is linked to poor OS in these TNBC patients [12]. Our KM plotter analyses further demonstrated that combined high expression of S100A7 (PSOR1) and SERPINE1 predicts significantly worse survival outcomes than either gene alone, with particularly strong effects in basal-like and IM TNBC, underscoring their clinical relevance for prognosis and therapeutic response. In this study, we reported that inhibition of S100A7 signaling was accompanied by enrichment of pro-inflammatory and antigen-presenting macrophage populations, suggesting a shift toward an anti-tumor immune microenvironment. In line with this observation, incorporation of macrophage-associated markers NOS2 or HLA-DRB significantly attenuated the adverse OS and RFS associated with high PSOR1/SERPINE1 expression in aggressive TNBC subtypes, indicating that macrophage activation status modulates PSOR1/SERPINE1-driven tumor aggressiveness. Moreover, our results indicate that targeting Serpin-E1 alongside Stat3 and RAGE inhibitors presents a promising avenue to enhance anti-metastatic effects and improve clinical outcomes in S100A7-expressing TNBC, especially among basal and IM subtype patients. This underscores the clinical relevance of targeting the S100A7/RAGE/Stat3/Serpin-E1 axis as a potential therapeutic strategy in TNBC.

In conclusion, this study elucidates the novel role of the S100A7/RAGE/Stat3/Serpin-E1 axis in driving tumor progression and immunosuppression in TNBC. In addition, identifying biomarkers such as S100A7 and Serpin-E1 also opens avenues for patient stratification and personalized treatment strategies for TNBC subtypes. Furthermore, the combinatorial treatment targeting the S100A7-mediated downstream signaling pathway paves the way for developing novel therapeutic strategies for TNBC patients with limited therapeutic options and the worst prognosis.

## Supplementary Information

Below is the link to the electronic supplementary material.

Supplemental Figure 1: **Analysis of S100A7 downregulation on Stat3 phosphorylation and effect of alone or combinatorial treatment of Stat3 and RAGE inhibitors on cell viability of S100A7 expressing TNBC cells**. (**A**). The cell lysates were harvested from Scramble (Scr) control and S100A7 knockdown (KD) MDA-MB-468 cells generated using an independent shRNA clone 2 and were analyzed for the level of S100A7, phosphorylation of activated phospho-Stat3 (Ser727 and Tyr705), total Stat3, and GAPDH. Cell viability assays of **B & C**). S100A7 overexpressing MDA-MB-231 and (**D & E**). S100A7-expressing MDA-MB-468 cells treated with vehicle controls (VC) or different concentrations of Stat3 and RAGE inhibitors. Cell viability assays of (**F**). S100A7 overexpressing MDA-MB-231 and (**G**). S100A7 expressing MDA-MB-468 cells treated with vehicle controls (VC) or combinations of different concentrations of Stat3 and RAGE inhibitors. Data are mean±SEM. (n= 3).

Supplemental Figure 2: **Impact of RAGE/Stat3 inhibition on S100A7-driven wound closure, in-vivo tumor cell proliferation, and total macrophage infiltration**. (**A**). Effects of Stat3 and RAGE inhibition on wound closure abilities of (**p**A). S100A7 overexpressing MDA-MB-231 cells, and (**B**). S100A7-expressing MDA-MB-468 cells (**C**). Immunofluorescence analysis of cancer cell proliferation marker (Ki-67) in tumor sections of S100A7 overexpressing bi-transgenic mice injected with MVT1 cells and treated with Stat3 and RAGE inhibitors alone or in combination. (**D**). Flow cytometric analysis of CD11b+F/80+ tumor-associated macrophages (TAMs) in tumor tissues of S100A7 overexpressing bi-transgenic mice injected with MVT1 cells and treated with vehicle control (VC), Stat3, or RAGE inhibitors alone or in combination. (n= 3). ****p< 0.0001; ns: non-significant.

Supplemental Figure 3: **RAGE/Stat3 inhibition reduces S100A7-driven TNBC by activating T-cell-mediated antitumor immunity**. The tumors harvested from S100A7-overexpression mice treated with vehicle control, RAGE, or Stat3 inhibitors, or their combination, were analyzed for the different immune cells using multi-color flow cytometry. Effects of alone or combined Stat3 and RAGE inhibition on abundance or infiltration of (**A**). Granzyme, IFNγ, and TNFα positive CD8+ T cells, as well as (**B**). CD44+, CD69+, and granzyme-positive CD4+ T cells. Data are mean±SEM. (n= 3). *p< 0.05; **p< 0.01; ***p< 0.001; ****p< 0.0001; ns: non-significant.

Supplemental Figure 4: **RAGE/Stat3 inhibition suppresses S100A7-driven TNBC by modulating CD4⁺ T cells and assessing the impact of T-cell depletion**. (**A**). The tumors harvested from S100A7-overexpression mice treated with vehicle control, RAGE, or Stat3 inhibitors, or their combination were analyzed for the different immune cells using multi-color flow cytometry. Effects of alone or combined Stat3 and RAGE inhibition on abundance or infiltration of IFNγ and TNFα positive CD4+ T cells. (**B**). Flow cytometry and bar plots depicting CD4⁺ and CD8⁺ T-cell depletion in Stat3/RAGE inhibitor–treated S100A7-overexpressing tumors. Data are mean±SEM. (n= 3 to 7). *p< 0.05; **p< 0.01; ***p< 0.001; ****p< 0.0001; ns: non-significant.

Supplemental Figure 5: **Expression of Serpin-E1 in breast tumor tissues and its correlation with S100A7 and immunosuppressive M2 macrophages**. Expression of the SERPINE1 gene was analyzed in normal and breast tumor tissues, including metastatic samples, using (**A**). GENT2 (normal = 475 and tumor = 5574), (**B**). GEPIA (normal = 291 and tumor = 1085), (**C**). TNMplot (normal = 242, tumor = 7569 and metastatic = 82), and (**D**). UALCAN databases. Expression of the SERPINE1 gene was analyzed in (**E**). Normal and different lymph nodes (normal = 114, N0 = 516, N1 = 362, N2 = 120, and N3 = 77) and (**F**). hormonal status of breast cancer patients analyzed by mining UALCAN and METABRIC databases. Analysis of the correlation of SERPINE1 gene expression with (**G**). S100A7 gene expression in different breast cancer types (n = 10930) and (**H**). M2 macrophage infiltration in the basal subtype of breast cancer (n=191). ****p< 0.0001.

Supplemental Figure 6: **Stat3 inhibition reduces S100A7-induced Serpin-E1, and combined Stat3/RAGE inhibition with Serpin-E1 neutralization suppresses metastasis in S100A7-high TNBC, with minimal effect in S100A7-negative tumors**. (**A**). Quantitation of Serpin-E1 in conditioned media of MDA-MB-231 scramble or vector control (231V) and S100A7 overexpressing MDA-MB-231 (S7OE) cell either treated with vehicle control (VC) or Stat3 inhibitor (IC50 value). Microscopic images show the H/E staining of (**B**). liver nodules, and (**C**). lung nodules in NSG mice injected with MDA-MB-231-S7OE cells and treated with Serpin-E1 nAb alone and in combination with Stat3 and RAGE inhibitors. (**D-F**). Representative harvested tumors, tumor volume, and weight in NSG mice injected with MDA-MB-231 cells that do not express S100A7 after the treatment of VC or Serpin-E1 nAb alone, RAGE/Stat3 inhibitors combination, and combination of Serpin-E1 nAb with RAGE/Stat3 inhibitors. Data are mean±SEM. (n= 3-4). ****p< 0.0001; ns: non-significant.

Supplementary Material 7.

Supplementary Material 8.

## Data Availability

All data generated during this study are included in this published article and its supplementary files.
