# Supplementary figures and images for: Therapeutic targeting of RAGE/STAT3 signaling abrogates S100A7-driven breast tumorigenicity and immune suppression
Source: Breast Cancer Res. 2026 Apr 29;28:112. doi: 10.1186/s13058-026-02281-0 (PMC13281475; doi:10.1186/s13058-026-02281-0)

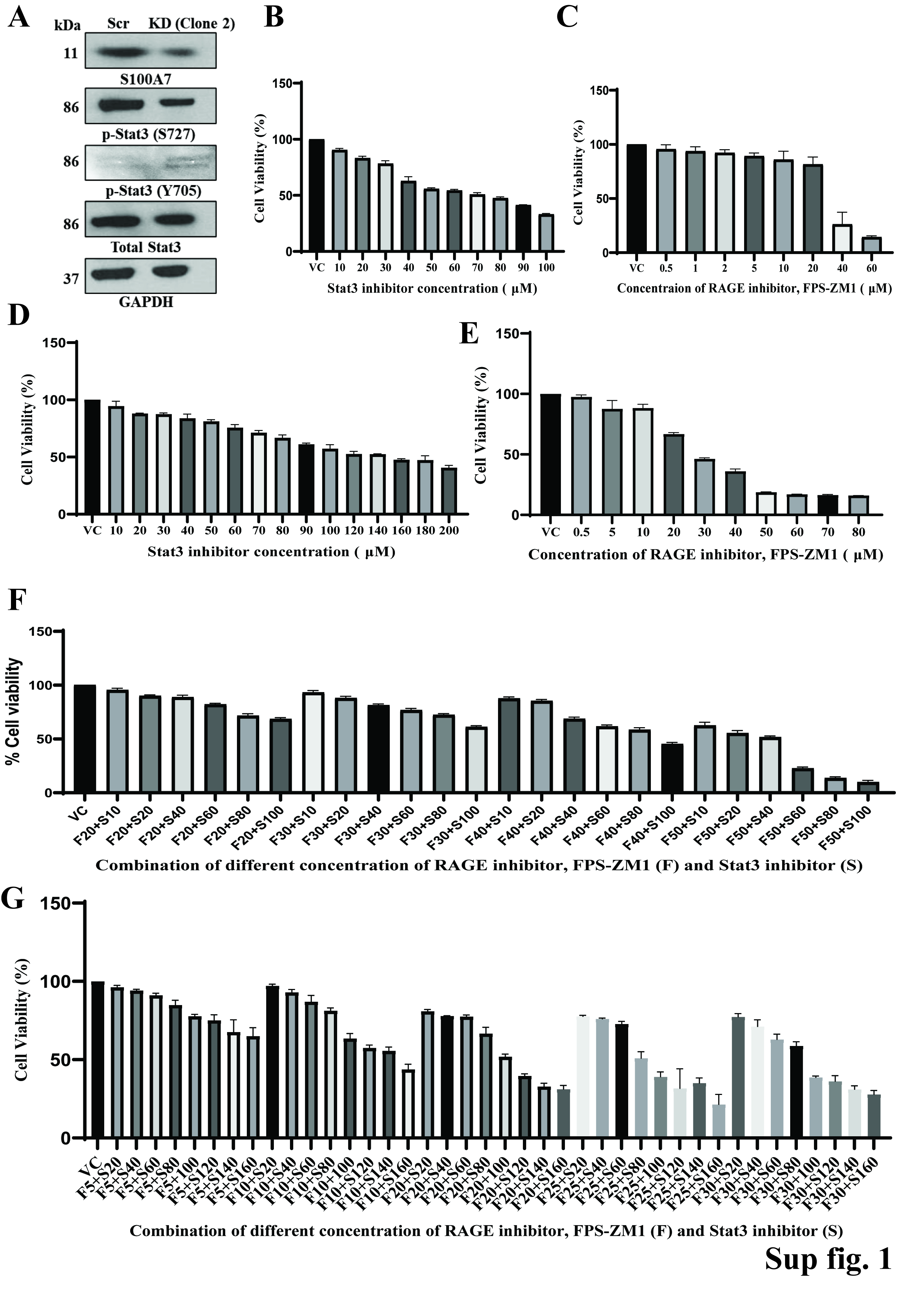

Supplement: Supplementary file 1 — Supplemental Figure 1: Analysis of S100A7 downregulation on Stat3 phosphorylation and effect of alone or combinatorial treatment of Stat3 and RAGE inhibitors on cell viability of S100A7 expressing TNBC cells. (A). The cell lysates were harvested from Scramble (Scr) control and S100A7 knockdown (KD) MDA-MB-468 cells generated using an independent shRNA clone 2 and were analyzed for the level of S100A7, phosphorylation of activated phospho-Stat3 (Ser727 and Tyr705), total Stat3, and GAPDH. Cell viability assays of B & C). S100A7 overexpressing MDA-MB-231 and (D & E). S100A7-expressing MDA-MB-468 cells treated with vehicle controls (VC) or different concentrations of Stat3 and RAGE inhibitors. Cell viability assays of (F). S100A7 overexpressing MDA-MB-231 and (G). S100A7 expressing MDA-MB-468 cells treated with vehicle controls (VC) or combinations of different concentrations of Stat3 and RAGE inhibitors. Data are mean±SEM. (n= 3). [file 13058_2026_2281_MOESM1_ESM.tif]

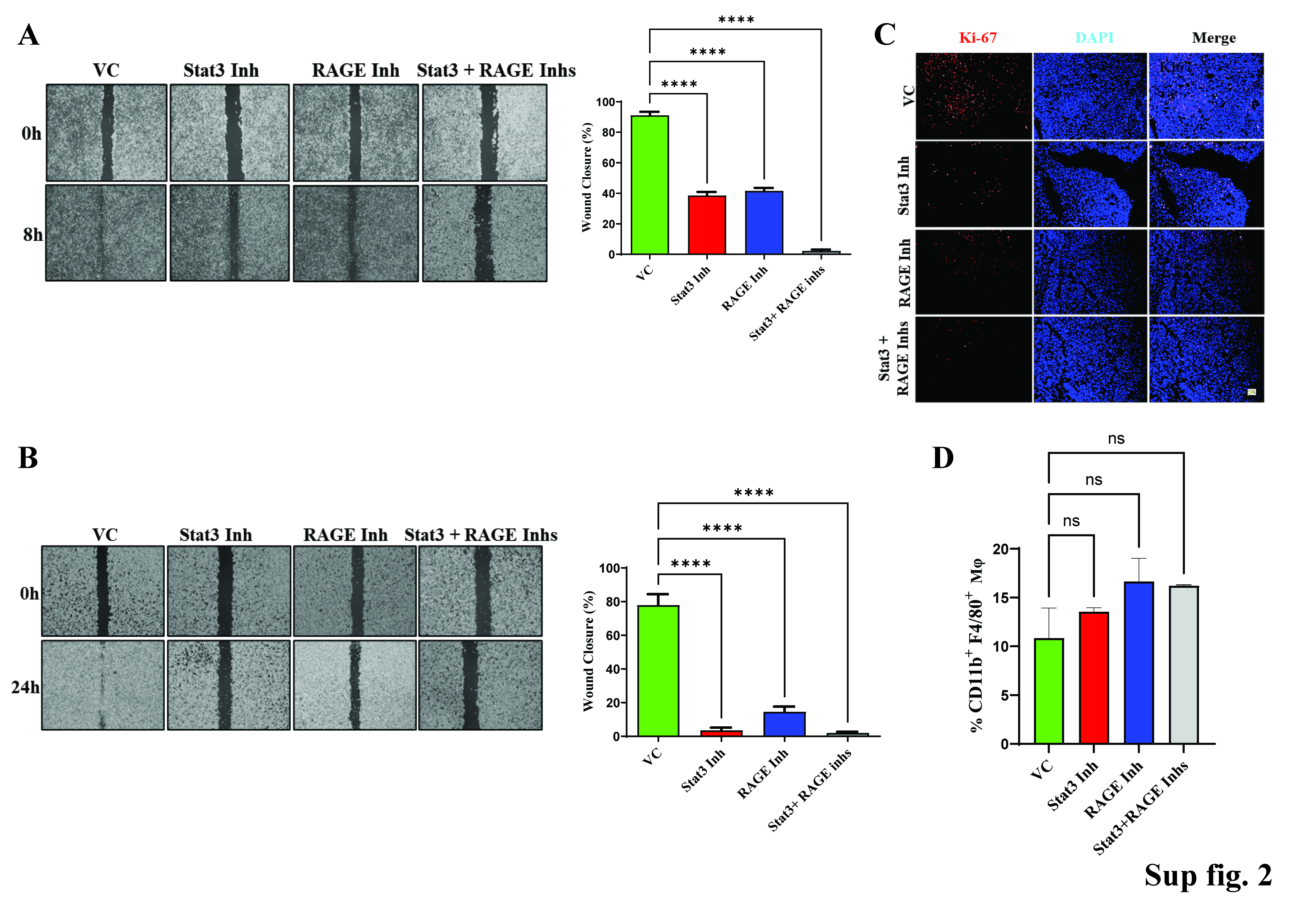

Supplement: Supplementary file 2 — Supplemental Figure 2: Impact of RAGE/Stat3 inhibition on S100A7-driven wound closure, in-vivo tumor cell proliferation, and total macrophage infiltration. (A). Effects of Stat3 and RAGE inhibition on wound closure abilities of (pA). S100A7 overexpressing MDA-MB-231 cells, and (B). S100A7-expressing MDA-MB-468 cells (C). Immunofluorescence analysis of cancer cell proliferation marker (Ki-67) in tumor sections of S100A7 overexpressing bi-transgenic mice injected with MVT1 cells and treated with Stat3 and RAGE inhibitors alone or in combination. (D). Flow cytometric analysis of CD11b+F/80+ tumor-associated macrophages (TAMs) in tumor tissues of S100A7 overexpressing bi-transgenic mice injected with MVT1 cells and treated with vehicle control (VC), Stat3, or RAGE inhibitors alone or in combination. (n= 3). ****p< 0.0001; ns: non-significant. [file 13058_2026_2281_MOESM2_ESM.tif]

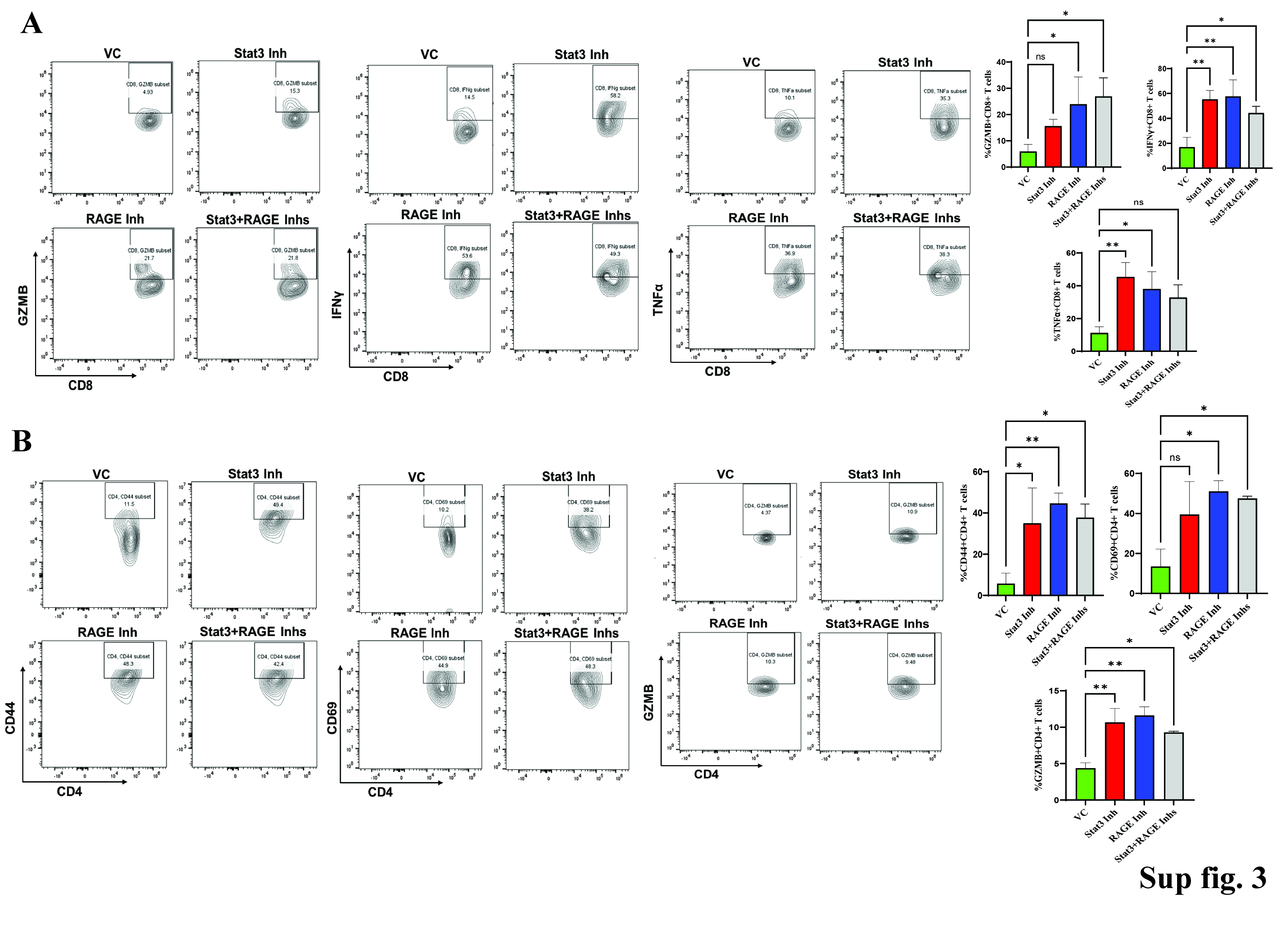

Supplement: Supplementary file 3 — Supplemental Figure 3: RAGE/Stat3 inhibition reduces S100A7-driven TNBC by activating T-cell-mediated antitumor immunity. The tumors harvested from S100A7-overexpression mice treated with vehicle control, RAGE, or Stat3 inhibitors, or their combination, were analyzed for the different immune cells using multi-color flow cytometry. Effects of alone or combined Stat3 and RAGE inhibition on abundance or infiltration of (A). Granzyme, IFNγ, and TNFα positive CD8+ T cells, as well as (B). CD44+, CD69+, and granzyme-positive CD4+ T cells. Data are mean±SEM. (n= 3). *p< 0.05; **p< 0.01; ***p< 0.001; ****p< 0.0001; ns: non-significant. [file 13058_2026_2281_MOESM3_ESM.tif]

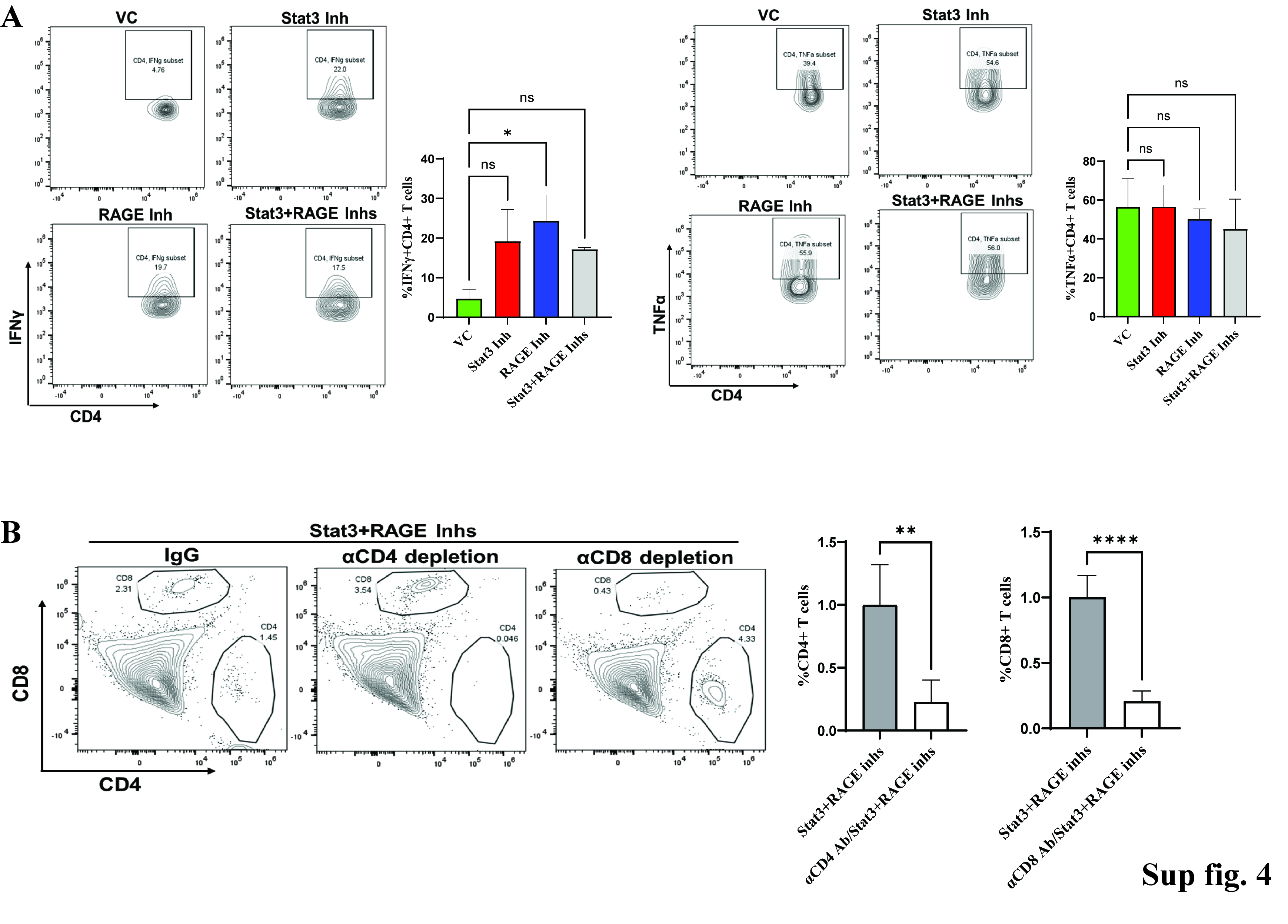

Supplement: Supplementary file 4 — Supplemental Figure 4: RAGE/Stat3 inhibition suppresses S100A7-driven TNBC by modulating CD4⁺ T cells and assessing the impact of T-cell depletion. (A). The tumors harvested from S100A7-overexpression mice treated with vehicle control, RAGE, or Stat3 inhibitors, or their combination were analyzed for the different immune cells using multi-color flow cytometry. Effects of alone or combined Stat3 and RAGE inhibition on abundance or infiltration of IFNγ and TNFα positive CD4+ T cells. (B). Flow cytometry and bar plots depicting CD4⁺ and CD8⁺ T-cell depletion in Stat3/RAGE inhibitor–treated S100A7-overexpressing tumors. Data are mean±SEM. (n= 3 to 7). *p< 0.05; **p< 0.01; ***p< 0.001; ****p< 0.0001; ns: non-significant. [file 13058_2026_2281_MOESM4_ESM.tif]

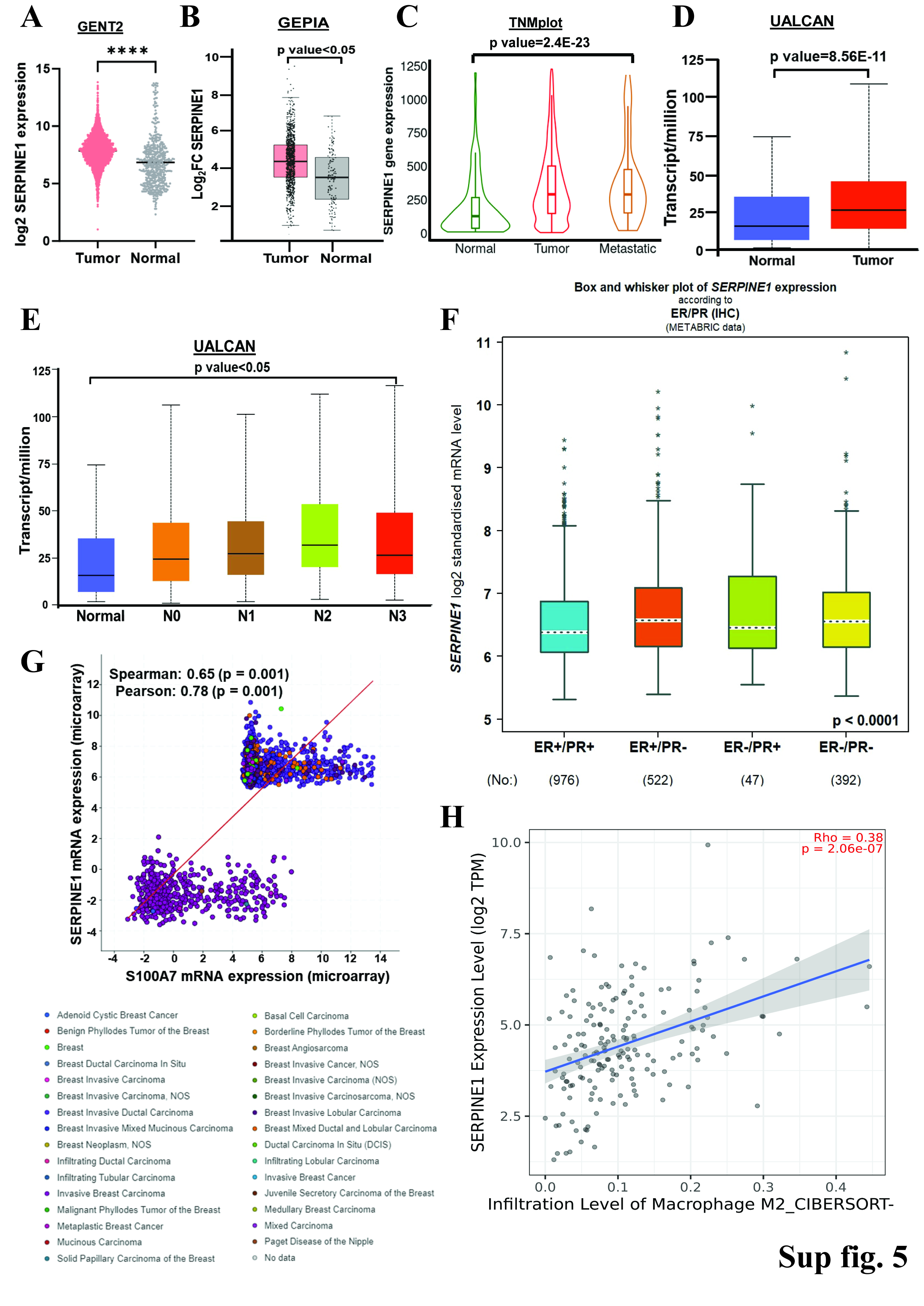

Supplement: Supplementary file 5 — Supplemental Figure 5: Expression of Serpin-E1 in breast tumor tissues and its correlation with S100A7 and immunosuppressive M2 macrophages. Expression of the SERPINE1 gene was analyzed in normal and breast tumor tissues, including metastatic samples, using (A). GENT2 (normal = 475 and tumor = 5574), (B). GEPIA (normal = 291 and tumor = 1085), (C). TNMplot (normal = 242, tumor = 7569 and metastatic = 82), and (D). UALCAN databases. Expression of the SERPINE1 gene was analyzed in (E). Normal and different lymph nodes (normal = 114, N0 = 516, N1 = 362, N2 = 120, and N3 = 77) and (F). hormonal status of breast cancer patients analyzed by mining UALCAN and METABRIC databases. Analysis of the correlation of SERPINE1 gene expression with (G). S100A7 gene expression in different breast cancer types (n = 10930) and (H). M2 macrophage infiltration in the basal subtype of breast cancer (n=191). ****p< 0.0001. [file 13058_2026_2281_MOESM5_ESM.tif]

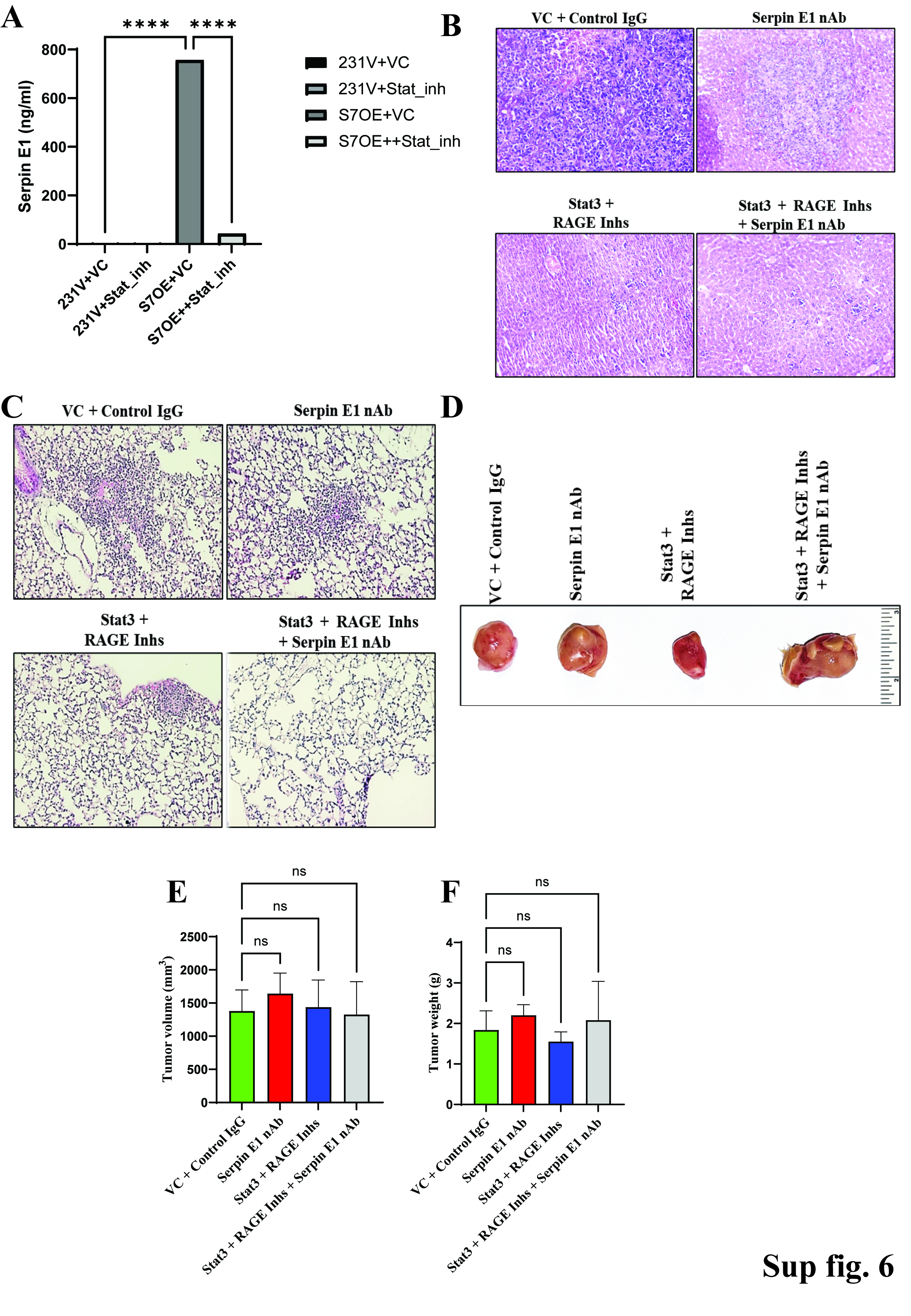

Supplement: Supplementary file 6 — Supplemental Figure 6: Stat3 inhibition reduces S100A7-induced Serpin-E1, and combined Stat3/RAGE inhibition with Serpin-E1 neutralization suppresses metastasis in S100A7-high TNBC, with minimal effect in S100A7-negative tumors. (A). Quantitation of Serpin-E1 in conditioned media of MDA-MB-231 scramble or vector control (231V) and S100A7 overexpressing MDA-MB-231 (S7OE) cell either treated with vehicle control (VC) or Stat3 inhibitor (IC50 value). Microscopic images show the H/E staining of (B). liver nodules, and (C). lung nodules in NSG mice injected with MDA-MB-231-S7OE cells and treated with Serpin-E1 nAb alone and in combination with Stat3 and RAGE inhibitors. (D-F). Representative harvested tumors, tumor volume, and weight in NSG mice injected with MDA-MB-231 cells that do not express S100A7 after the treatment of VC or Serpin-E1 nAb alone, RAGE/Stat3 inhibitors combination, and combination of Serpin-E1 nAb with RAGE/Stat3 inhibitors. Data are mean±SEM. (n= 3-4). ****p< 0.0001; ns: non-significant. [file 13058_2026_2281_MOESM6_ESM.tif]
