# Supplementary material for: Therapeutic targeting of RAGE/STAT3 signaling abrogates S100A7-driven breast tumorigenicity and immune suppression
Source: Breast Cancer Res. 2026 Apr 29;28:112. doi: 10.1186/s13058-026-02281-0 (PMC13281475; doi:10.1186/s13058-026-02281-0)
Supplement: Supplementary file 7 — Supplementary Material 7. [file 13058_2026_2281_MOESM7_ESM.docx]

**Supplemental figures**

**Supplemental Figure 1: Analysis of S100A7 downregulation on Stat3 phosphorylation and effect of alone or combinatorial treatment of Stat3 and RAGE inhibitors on cell viability of S100A7 expressing TNBC cells. (A).** The cell lysates were harvested from Scramble (Scr) control and S100A7 knockdown (KD) MDA-MB-468 cells generated using an independent shRNA clone 2 and were analyzed for the level of S100A7, phosphorylation of activated phospho-Stat3 (Ser727 and Tyr705), total Stat3, and GAPDH. Cell viability assays of **(B & C).** S100A7 overexpressing MDA-MB-231 and **(D & E).** S100A7-expressing MDA-MB-468 cells treated with vehicle controls (VC) or different concentrations of Stat3 and RAGE inhibitors. Cell viability assays of **(F).** S100A7 overexpressing MDA-MB-231 and **(G).** S100A7 expressing MDA-MB-468 cells treated with vehicle controls (VC) or combinations of different concentrations of Stat3 and RAGE inhibitors. Data are mean±SEM. (n= 3).

**Supplemental Figure 2: Impact of RAGE/Stat3 inhibition on S100A7-driven wound closure, *in-vivo* tumor cell proliferation, and total macrophage infiltration. (A).** Effects of Stat3 and RAGE inhibition on wound closure abilities of **(A).** S100A7 overexpressing MDA-MB-231 cells, and **(B).** S100A7-expressing MDA-MB-468 cells **(C).** Immunofluorescence analysis of cancer cell proliferation marker (Ki-67) in tumor sections of S100A7 overexpressing bi-transgenic mice injected with MVT1 cells and treated with Stat3 and RAGE inhibitors alone or in combination. **(D).** Flow cytometric analysis of CD11b^+^F/80^+^ tumor-associated macrophages (TAMs) in tumor tissues of S100A7 overexpressing bi-transgenic mice injected with MVT1 cells and treated with vehicle control (VC), Stat3, or RAGE inhibitors alone or in combination. (n= 3-7). ****p<0.0001; ns: non-significant.

**Supplemental Figure 3: RAGE/Stat3 inhibition reduces S100A7-driven TNBC by activating T-cell-mediated antitumor immunity.** The tumors harvested from S100A7-overexpression mice treated with vehicle control, RAGE, or Stat3 inhibitors, or their combination, were analyzed for the different immune cells using multi-color flow cytometry. Effects of alone or combined Stat3 and RAGE inhibition on abundance or infiltration of **(A).** Granzyme, IFNγ, and TNFα positive CD8^+^ T cells, as well as **(B).** CD44^+^, CD69^+,^ and granzyme-positive CD4^+^ T cells. Data are mean±SEM. (n= 3 to 7). *p<0.05; **p<0.01; ***p<0.001; ****p<0.0001; ns: non-significant.

**Supplemental Figure 4: RAGE/Stat3 inhibition suppresses S100A7-driven TNBC by modulating CD4⁺ T cells and assessing the impact of T-cell depletion. (A).** The tumors harvested from S100A7-overexpression mice treated with vehicle control, RAGE, or Stat3 inhibitors, or their combination were analyzed for the different immune cells using multi-color flow cytometry. Effects of alone or combined Stat3 and RAGE inhibition on abundance or infiltration of IFNγ and TNFα positive CD4^+^ T cells. **(B).** Flow cytometry and bar plots depicting CD4⁺ and CD8⁺ T-cell depletion in Stat3/RAGE inhibitor–treated S100A7-overexpressing tumors. Data are mean±SEM. (n= 3 to 7). *p<0.05; **p<0.01; ***p<0.001; ****p<0.0001; ns: non-significant.

**Supplemental Figure 5: Expression of Serpin-E1 in breast tumor tissues and its correlation with S100A7 and immunosuppressive M2 macrophages.** Expression of the SERPINE1 gene was analyzed in normal and breast tumor tissues, including metastatic samples, using **(A).** GENT2 (normal = 475 and tumor = 5574), **(B).** GEPIA (normal = 291 and tumor = 1085), **(C).** TNMplot (normal = 242, tumor = 7569 and metastatic = 82), and **(D).** UALCAN databases. Expression of the SERPINE1 gene was analyzed in **(E).** Normal and different lymph nodes (normal = 114, N0 = 516, N1 = 362, N2 = 120, and N3 = 77) and **(F).** hormonal status of breast cancer patients analyzed by mining UALCAN and METABRIC databases. Analysis of the correlation of SERPINE1 gene expression with **(G).** S100A7 gene expression in different breast cancer types (n = 10930) and **(H).** M2 macrophage infiltration in the basal subtype of breast cancer (n=191). ****p<0.0001.

**Supplemental Figure 6: Stat3 inhibition reduces S100A7-induced Serpin-E1, and combined Stat3/RAGE inhibition with Serpin-E1 neutralization suppresses metastasis in S100A7-high TNBC, with minimal effect in S100A7-negative tumors. (A).** Quantitation of Serpin-E1 in conditioned media of MDA-MB-231 scramble or vector control (231V) and S100A7 overexpressing MDA-MB-231 (S7OE) cell either treated with vehicle control (VC) or Stat3 inhibitor (IC_50_ value). Microscopic images show the H/E staining of **(B).** liver nodules, and **(C).** lung nodules in NSG mice injected with MDA-MB-231-S7OE cells and treated with Serpin-E1 nAb alone and in combination with Stat3 and RAGE inhibitors. **(D-F).** Representative harvested tumors, tumor volume, and weight in NSG mice injected with MDA-MB-231 cells that do not express S100A7 after the treatment of VC or Serpin-E1 nAb alone, RAGE/Stat3 inhibitors combination, and combination of Serpin-E1 nAb with RAGE/Stat3 inhibitors. Data are mean±SEM. (n= 3-4). ****p<0.0001; ns: non-significant.
