# Supplementary material for: Therapeutic targeting of RAGE/STAT3 signaling abrogates S100A7-driven breast tumorigenicity and immune suppression
Source: Breast Cancer Res. 2026 Apr 29;28:112. doi: 10.1186/s13058-026-02281-0 (PMC13281475; doi:10.1186/s13058-026-02281-0)
Supplement: Supplementary file 8 — Supplementary Material 8. [file 13058_2026_2281_MOESM8_ESM.pdf]

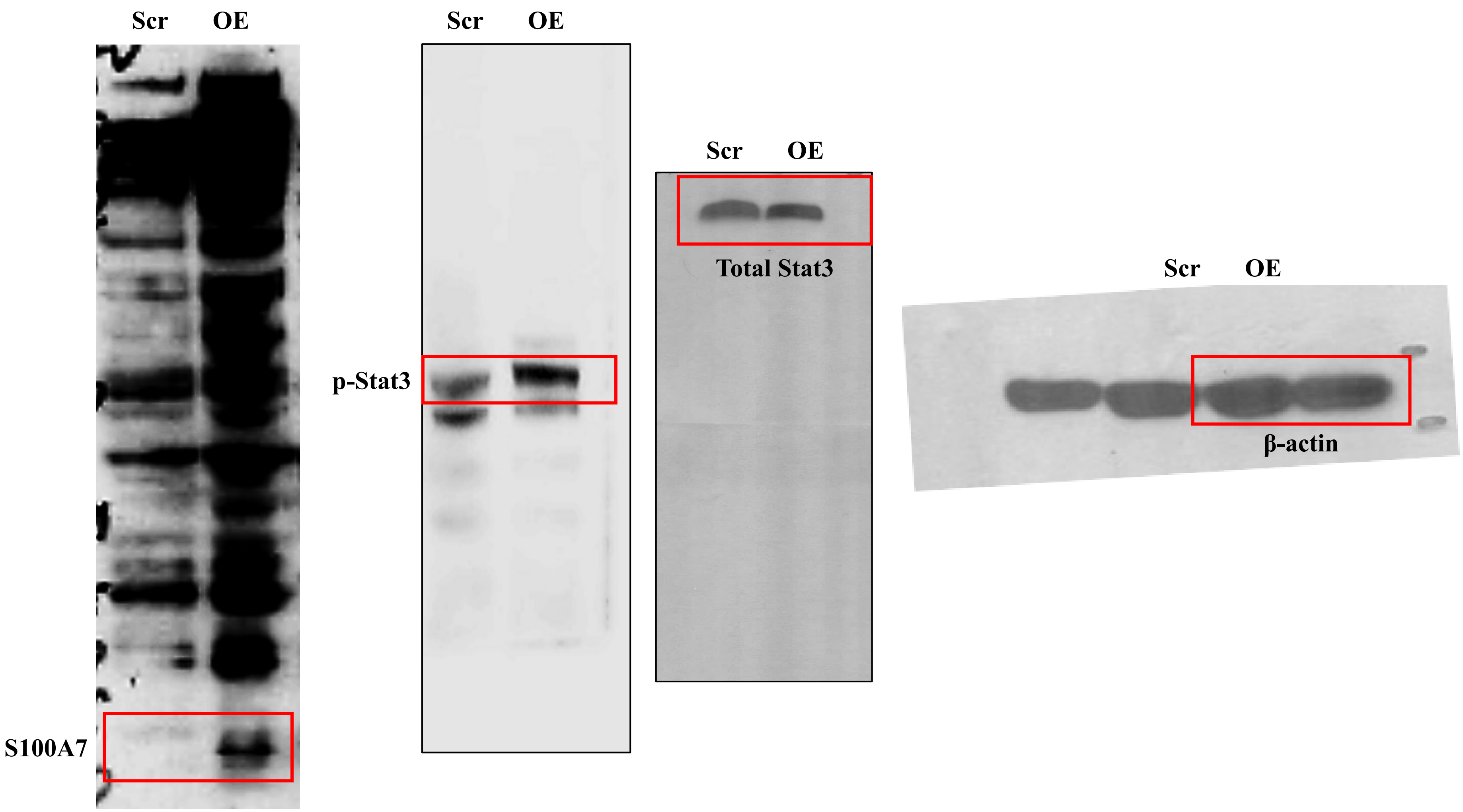

Fig. 1A

Note: Cropped blots are highlighted in red box

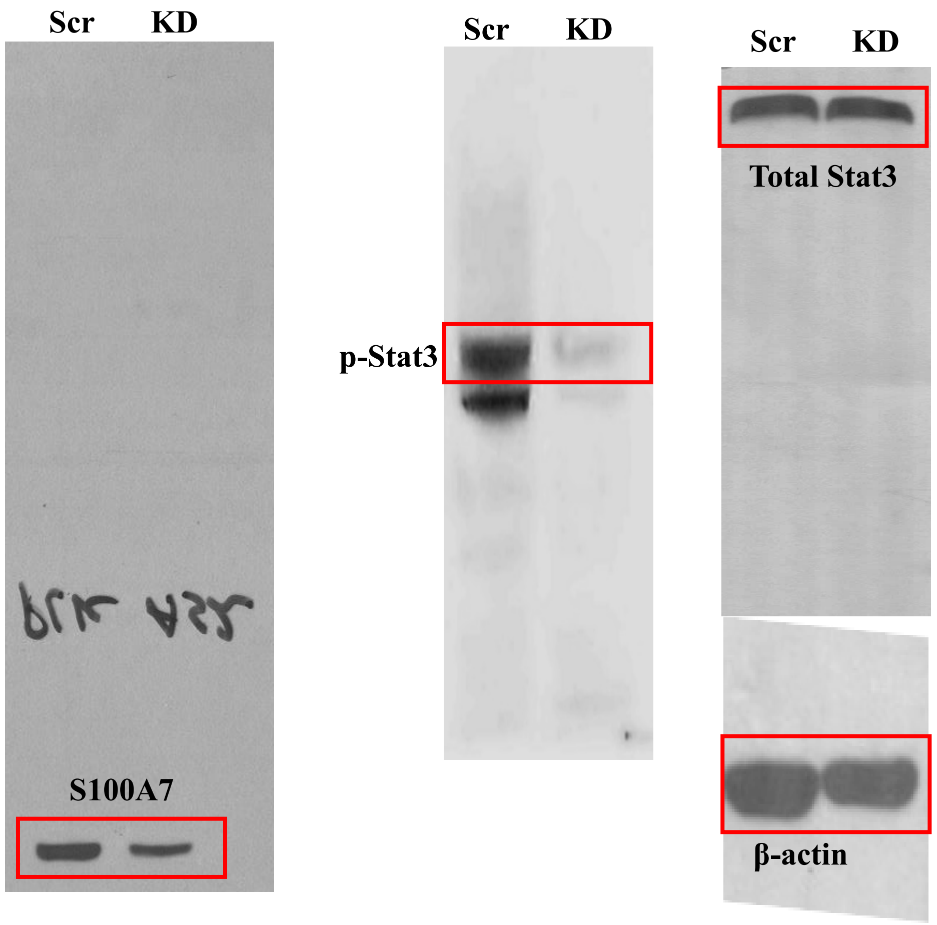

Fig. 1B

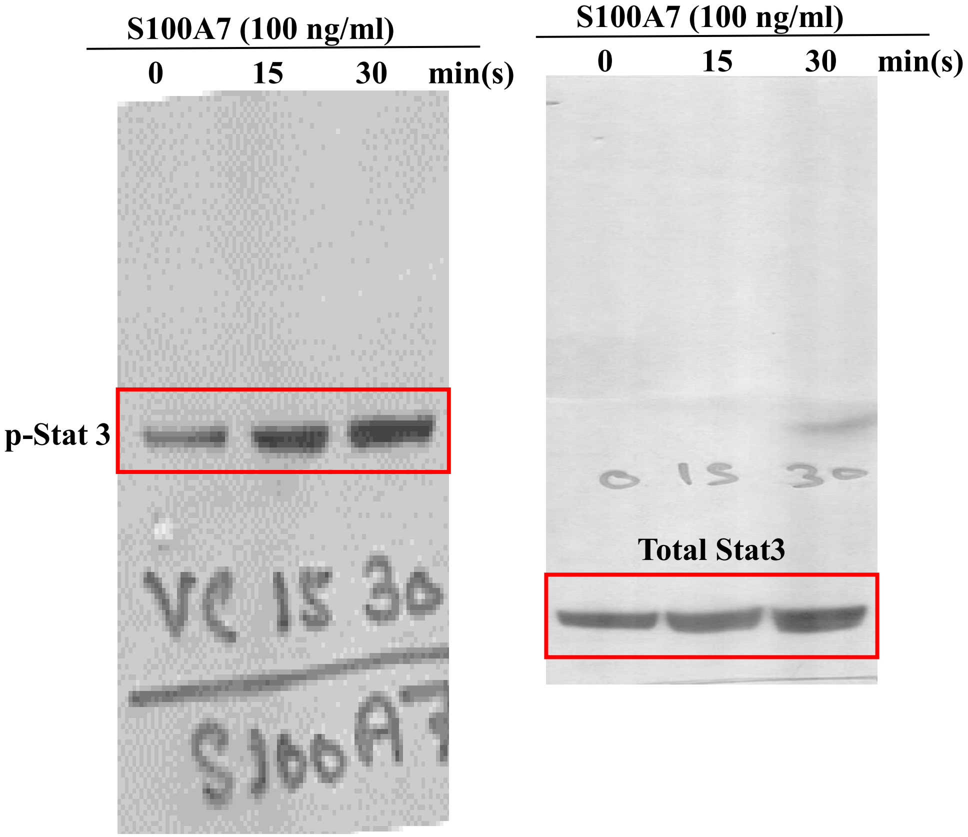
S100A7 (100 ng/ml)
0 15 30 min(s)
p-Stat 3
S100A7 (100 ng/ml)
0 15 30 min(s)
Total Stat3

Fig. 1C

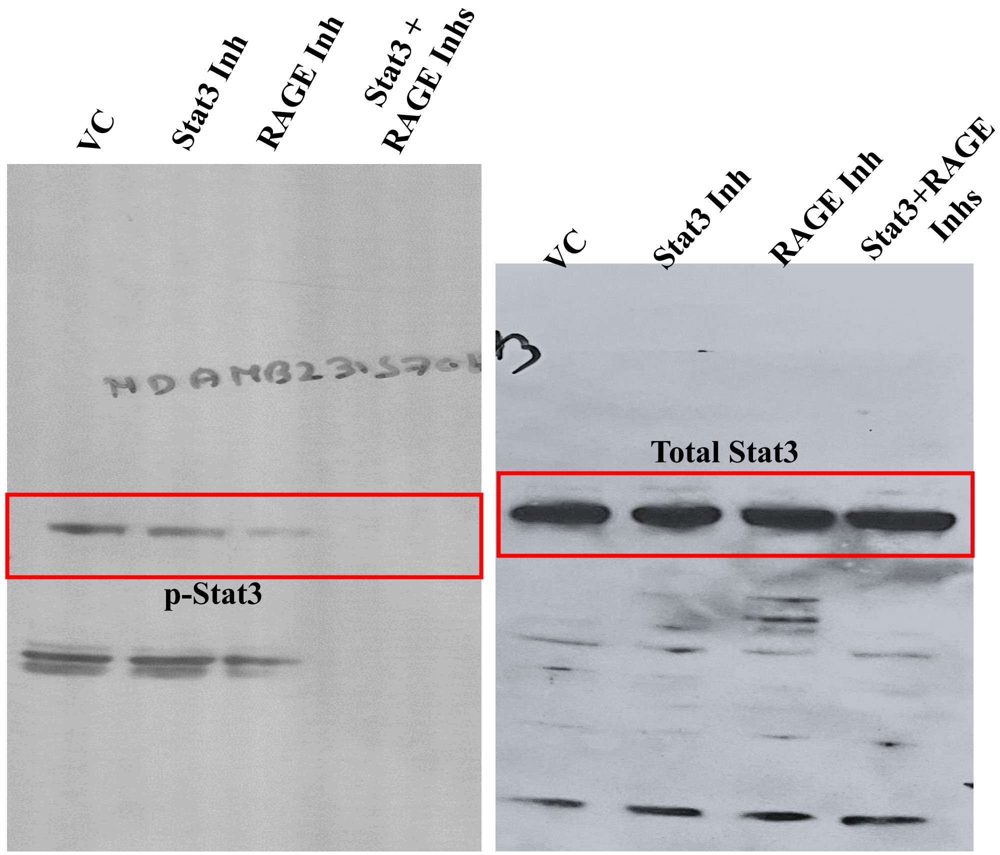
VC
Stat3 Inh
RAGE Inh
Stat3 + RAGE Inhs
p-Stat3
VC
Stat3 Inh
RAGE Inh
Stat3+RAGE Inhs
Total Stat3

Fig. 1F

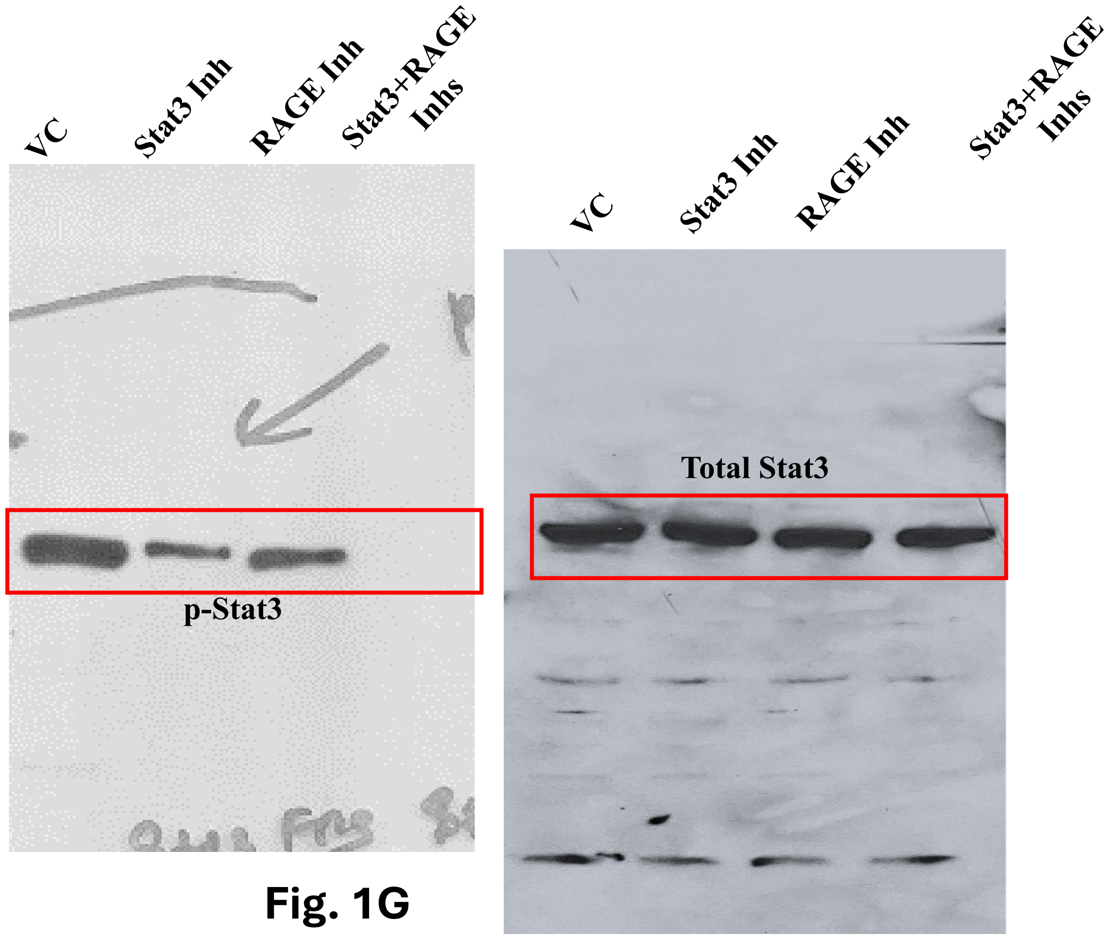
VC
Stat3 Inh
RAGE Inh
Stat3+RAGE Inhs
p-Stat3
VC
Stat3 Inh
RAGE Inh
Stat3+RAGE Inhs
Total Stat3

Fig. 1G

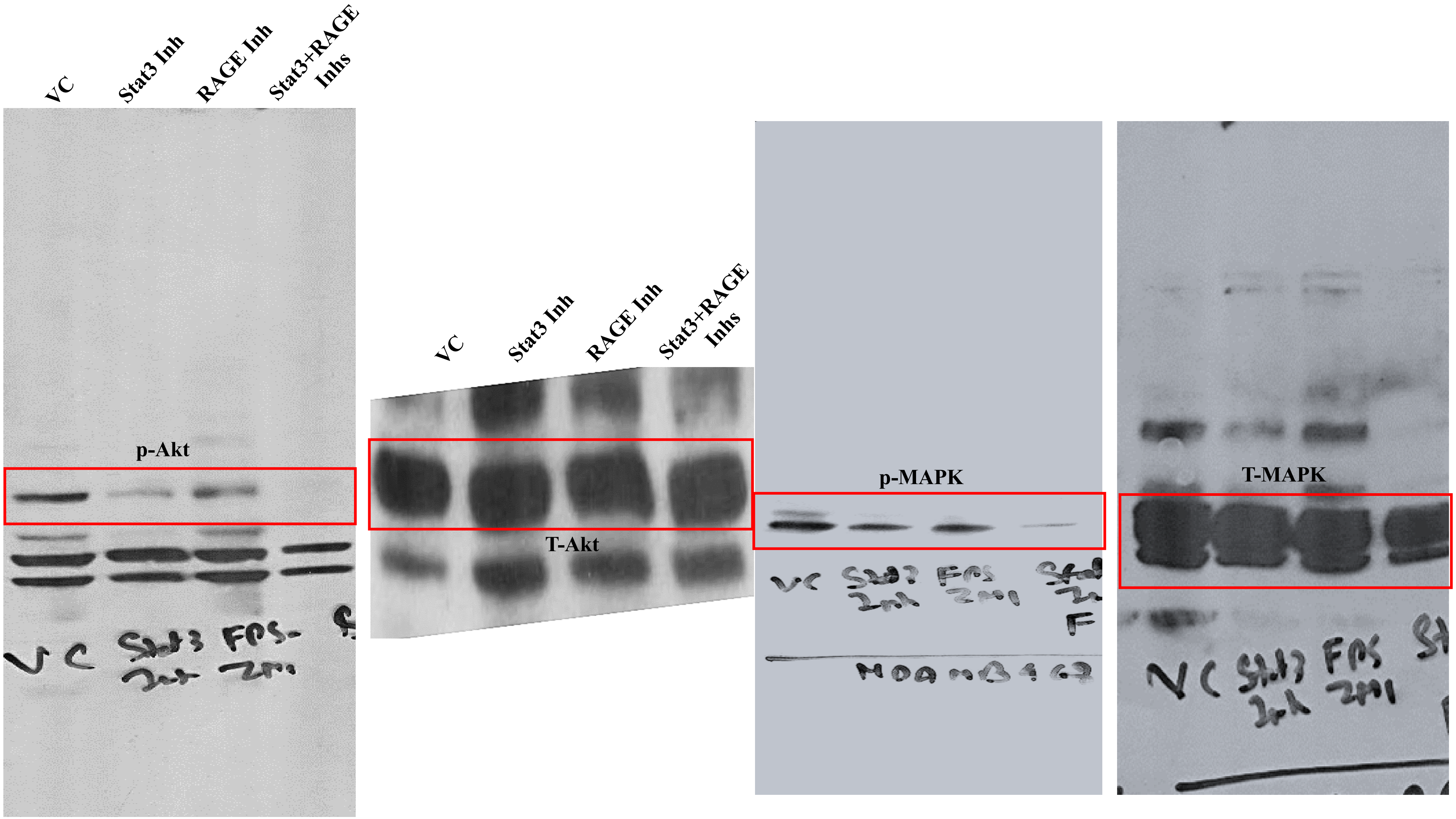
VC
Stat3 Inh
RAGE Inh
Stat3+RAGE Inhs
p-Akt
VC
Stat3 Inh
RAGE Inh
Stat3+RAGE Inhs
T-Akt
p-MAPK
T-MAPK

Fig. 2A

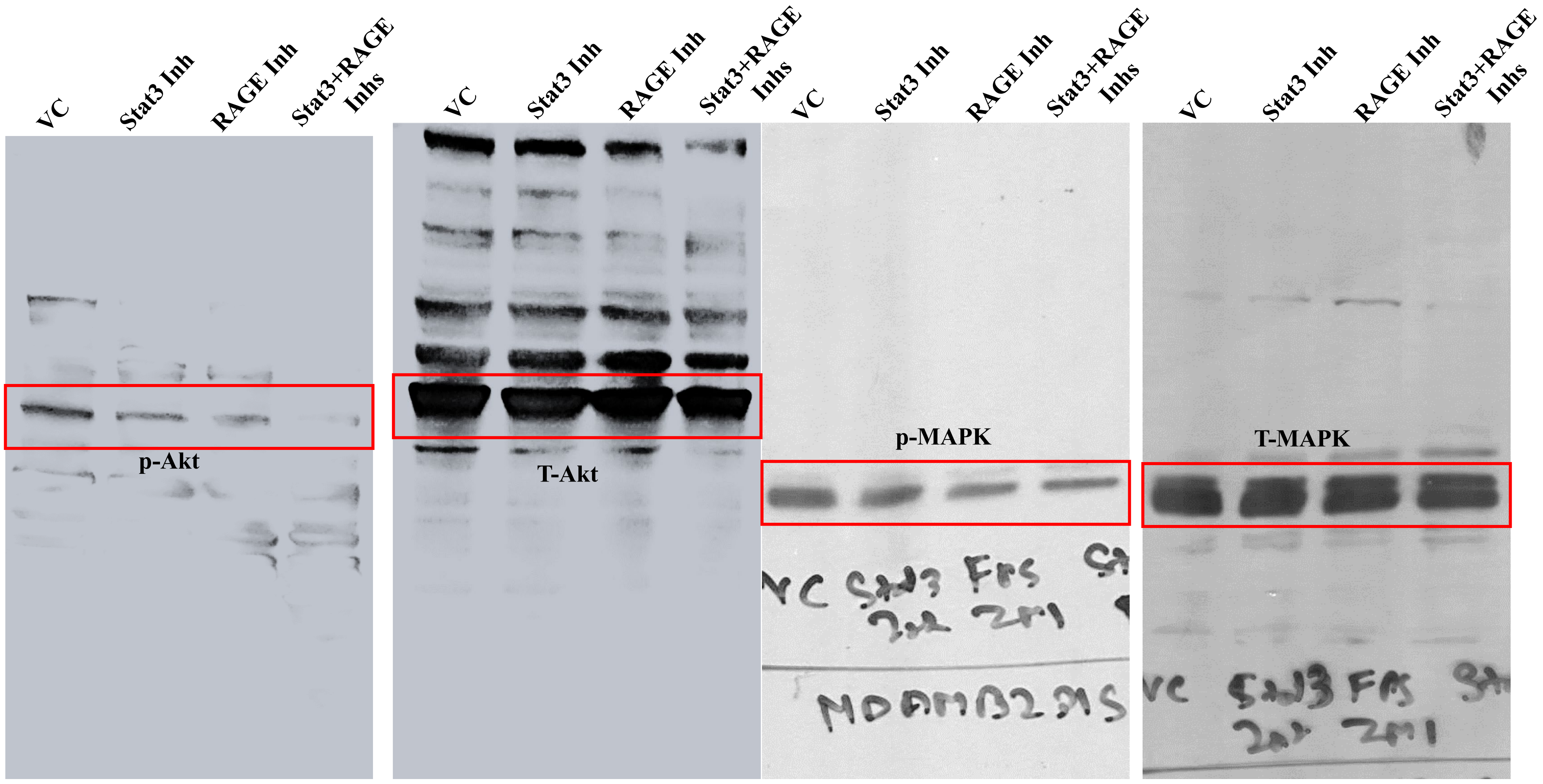

Fig. 2B

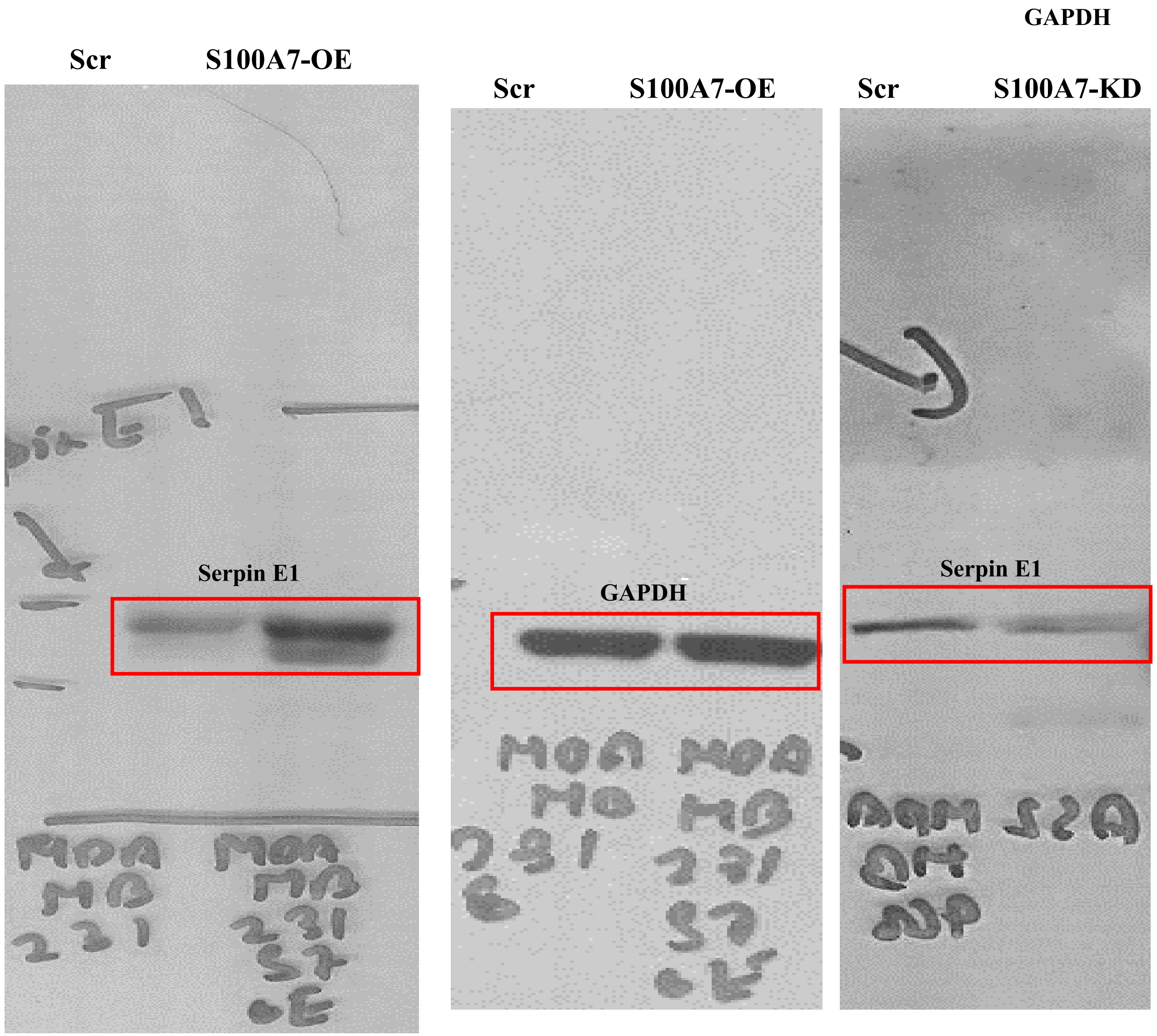

Fig. 6C

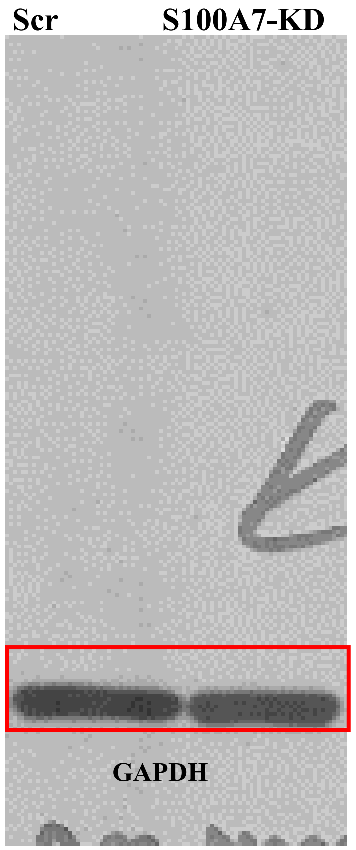

Fig. 6D

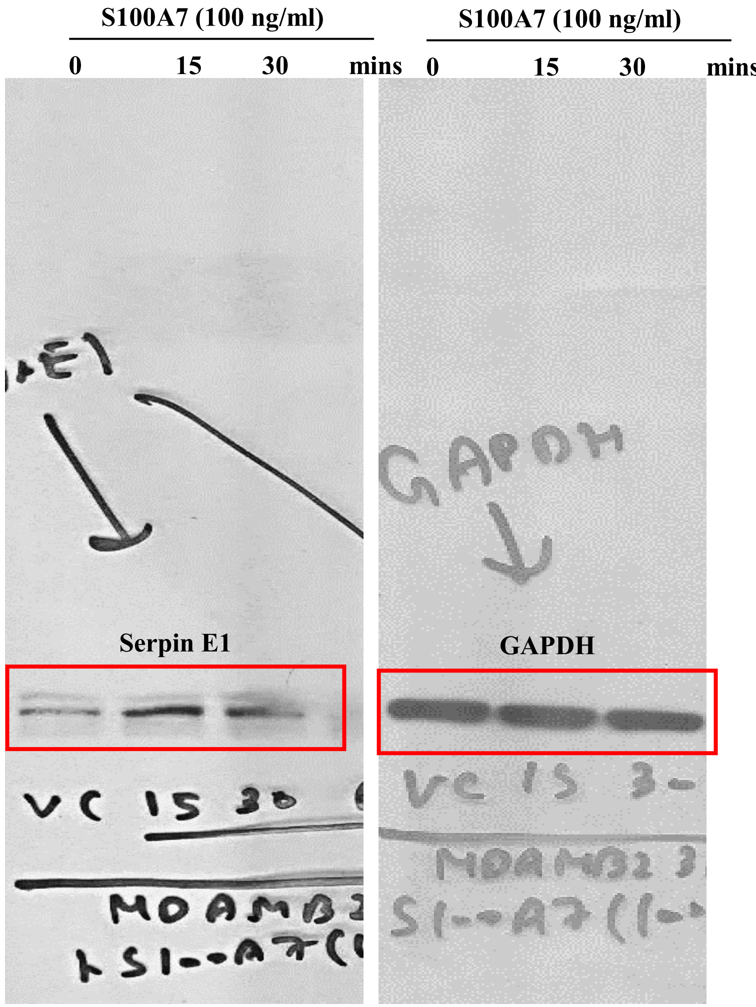

Fig. 6E

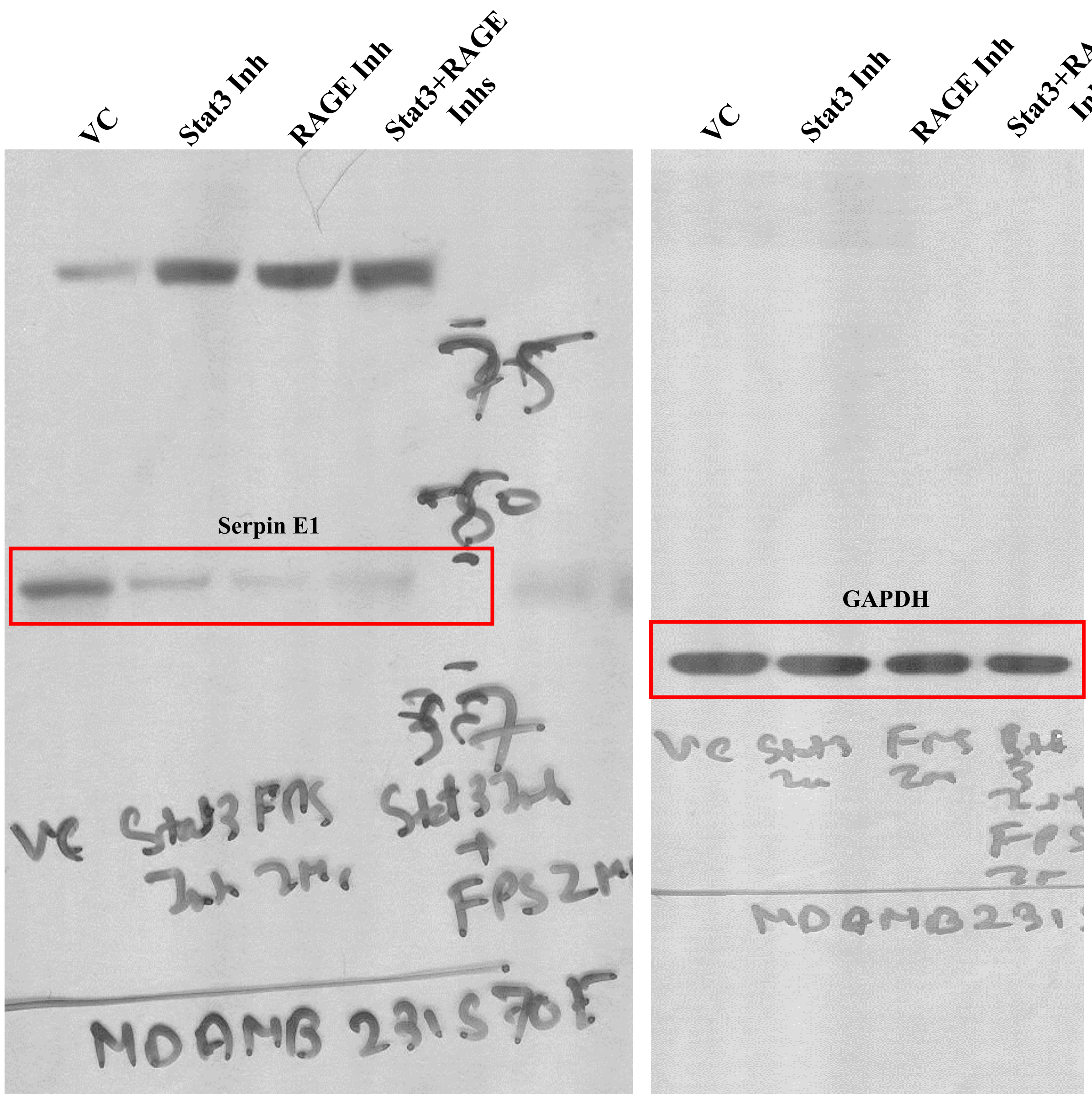
VC
Stat3 Inh
RAGE Inh
Stat3+RAGE Inhs
Serpin E1
GAPDH

Fig. 6F

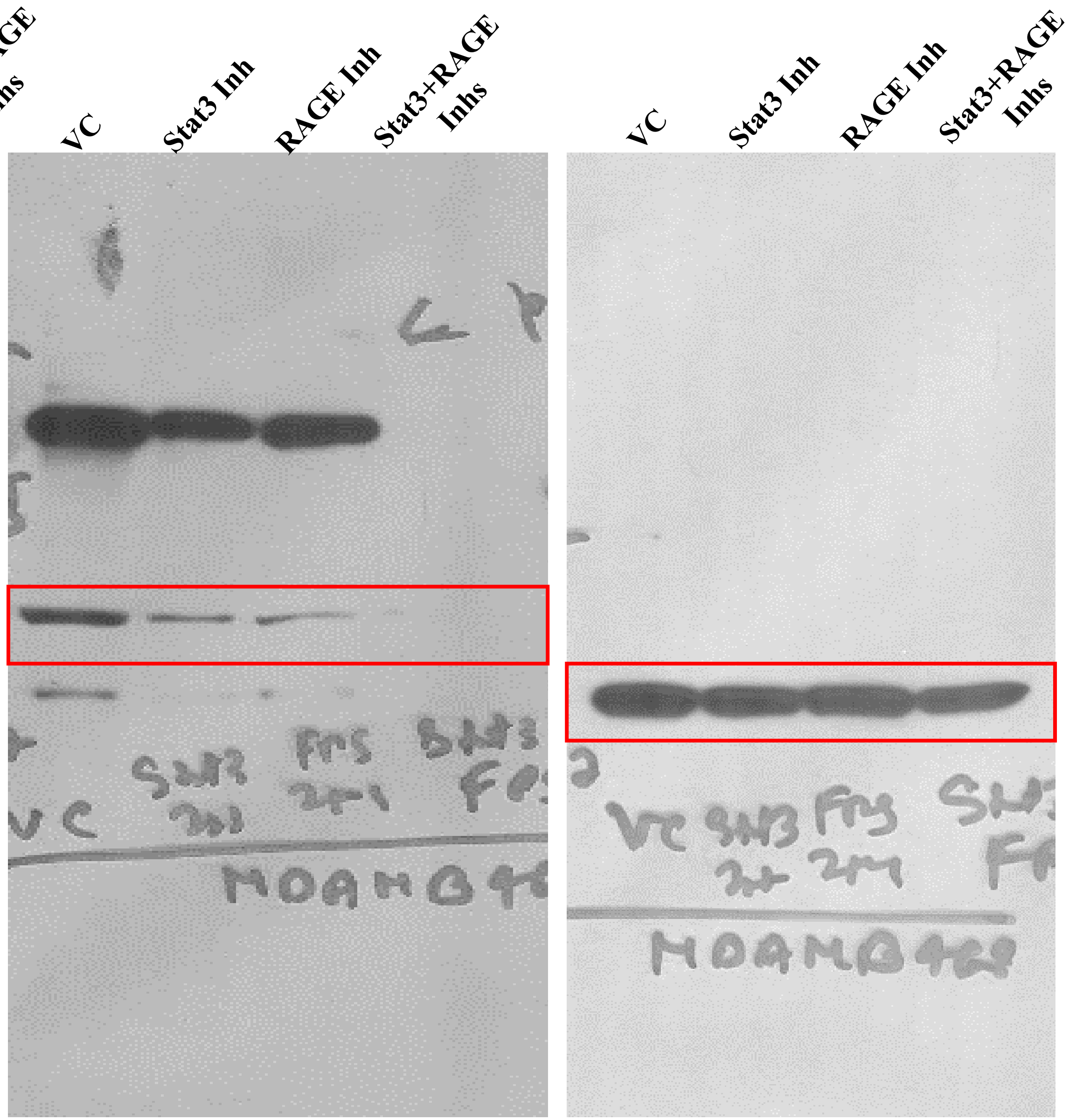
VC
Stat3 Inh
RAGE Inh
Stat3+RAGE Inhs

Fig. 6G

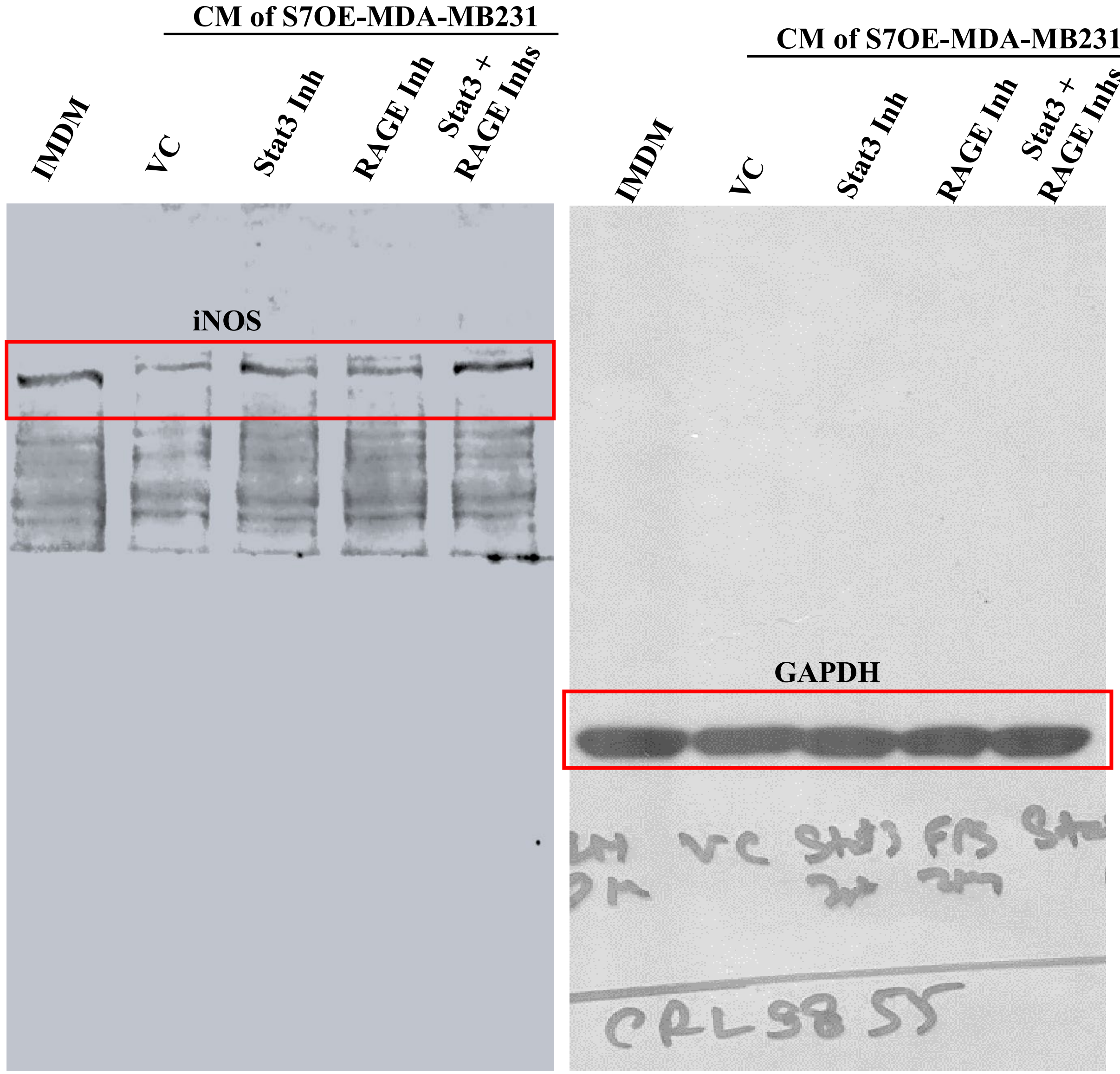
CM of S7OE-MDA-MB231
IMDM
VC
Stat3 Inh
RAGE Inh
Stat3 + RAGE Inhs
iNOS
CM of S7OE-MDA-MB231
IMDM
VC
Stat3 Inh
RAGE Inh
Stat3 + RAGE Inhs
GAPDH

Fig. 7A

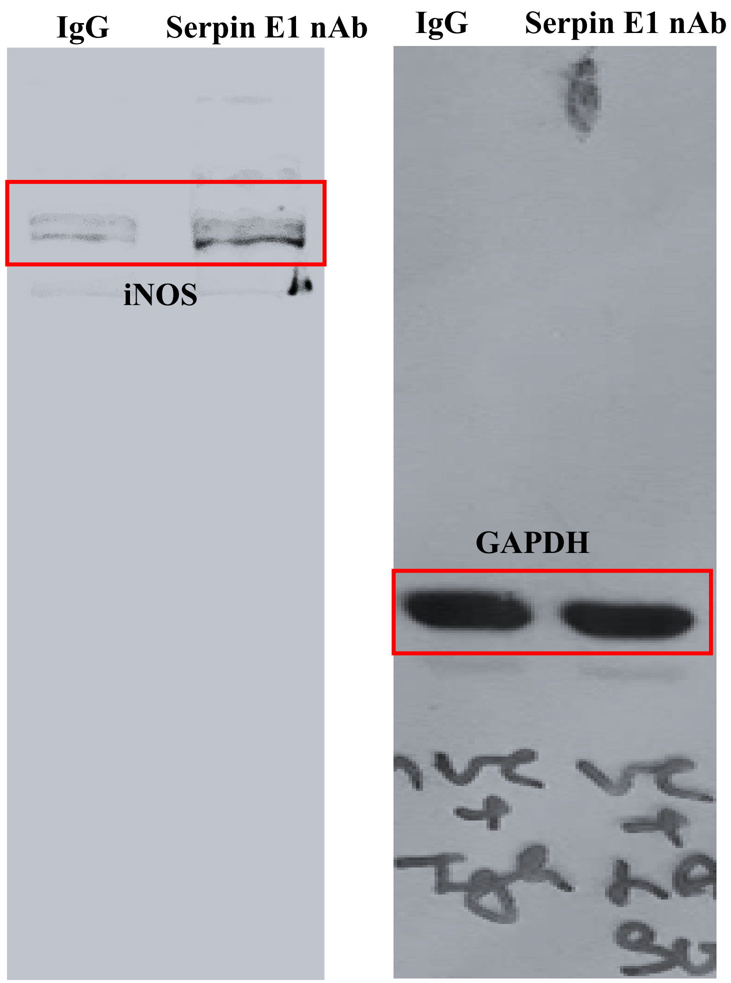
IgG
Serpin E1 nAb
iNOS
IgG
Serpin E1 nAb
GAPDH

Fig. 7B
